# Organic acid cross-linked 3D printed cellulose nanocomposite bioscaffolds with controlled porosity, mechanical strength, and biocompatibility

**DOI:** 10.1016/j.isci.2022.104263

**Published:** 2022-04-16

**Authors:** Andreja Dobaj Štiglic, Fazilet Gürer, Florian Lackner, Doris Bračič, Armin Winter, Lidija Gradišnik, Damjan Makuc, Rupert Kargl, Isabel Duarte, Janez Plavec, Uros Maver, Marco Beaumont, Karin Stana Kleinschek, Tamilselvan Mohan

**Affiliations:** 1 University of Maribor, Faculty of Mechanical Engineering, Laboratory for Characterization and Processing of Polymers, Smetanova Ulica 17, 2000 Maribor, Slovenia; 2 Graz University of Technology, Institute for Chemistry and Technology of Biobased System (IBioSys), Stremayrgasse 9, 8010 Graz, Austria; 3 University of Natural Resources and Life Sciences (BOKU) Institute of Wood Technology and Renewable Materials, Konrad-Lorenz-Strasse 24, 3430 Tulln, Austria; 4 University of Maribor, Faculty of Medicine, Institute of Biomedical Sciences, Taborska Ulica 8, 2000 Maribor, Slovenia; 5 Slovenian NMR Center, National Institute of Chemistry, Hajdrihova 19, 1001 Ljubljana, Slovenia; 6 University of Aveiro, Department of Mechanical Engineering, Center for Mechanical Technology and Automation, Campus Universitário de Santiago, 3810-193 Aveiro, Portugal; 7 EN→FIST Center of Excellence, Trg OF 13, 1000 Ljubljana, Slovenia; 8 University of Ljubljana, Faculty of Chemistry and Chemical Technology, Večna pot 113, 1000 Ljubljana, Slovenia; 9 University of Natural Resources and Life Sciences (BOKU), Institute of Chemistry of Renewable Resources, Konrad-Lorenz-Strasse 24, 3430 Tulln, Austria

**Keywords:** Tissue Engineering, Materials science, Biomaterials

## Abstract

Herein, we fabricated chemically cross-linked polysaccharide-based three-dimensional (3D) porous scaffolds using an ink composed of nanofibrillated cellulose, carboxymethyl cellulose, and citric acid (CA), featuring strong shear thinning behavior and adequate printability. Scaffolds were produced by combining direct-ink-writing 3D printing, freeze-drying, and dehydrothermal heat-assisted cross-linking techniques. The last step induces a reaction of CA. Degree of cross-linking was controlled by varying the CA concentration (2.5–10.0 wt.%) to tune the structure, swelling, degradation, and surface properties (pores: 100-450 μm, porosity: 86%) of the scaffolds in the dry and hydrated states. Compressive strength, elastic modulus, and shape recovery of the cross-linked scaffolds increased significantly with increasing cross-linker concentration. Cross-linked scaffolds promoted clustered cell adhesion and showed no cytotoxic effects as determined by the viability assay and live/dead staining with human osteoblast cells. The proposed method can be extended to all polysaccharide-based materials to develop cell-friendly scaffolds suitable for tissue engineering applications.

## Highlights

• Chemically cross-linked polysaccharide-based 3D porous scaffolds were fabricated
• Physicochemical and mechanical properties increased with cross-linker concentration
• Lower cross-linker concentration led to higher porosity and interconnected pores
• Scaffolds promoted clustered cell adhesion and showed no cytotoxic effects

## Graphical abstract

**Figure:**
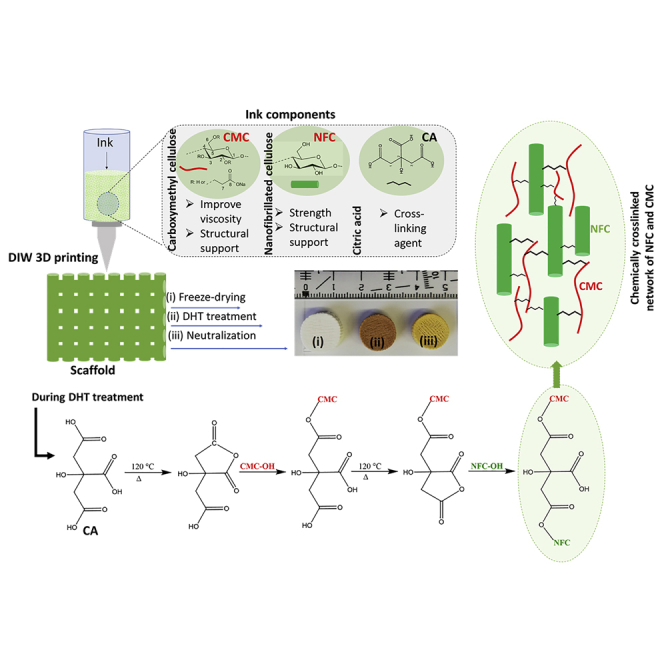

## Introduction

Lately, there has been an increasing interest in the use of renewable biopolymers for the development of low-cost three-dimensional (3D) scaffolds for soft and hard tissue engineering (TE) applications (Kahl et al., 2019; Li et al., 2020). The 3D porous morphology and interconnected pores of the scaffold were found to accelerate the adhesion, migration, and proliferation of cells and eventually tissue ingrowth (Abbasi et al., 2020; Li et al., 2021), and such a porous scaffold design is very crucial for efficient tissue regeneration and repair. This explains the current efforts to prepare scaffolds with controllable properties, such as shape, porosity, surface morphology, chemistry, degradation, and mechanical stability (Williams, 2019). To obtain such well-designed 3D porous scaffolds with the aforementioned properties is still a difficult task to achieve by traditional methods such as foaming and casting techniques.

To mimic the 3D architecture of cellular/tissue microenvironments and recapitulate biological functions, direct-ink-writing (DIW) 3D printing has proven to be versatile method that enables the fabrication of customized TE scaffolds with highly ordered porous morphology, structure, and function (Bose et al., 2013). Besides the adequate porosity and interconnected pore size, high printability, shape fidelity, and mechanical stability of the scaffolds are essential properties for the inks used in DIW 3D printing (Mohan et al., 2020). So far, many combinations of natural and synthetic polymers and inorganic materials have been investigated to develop inks and new types of 3D porous scaffolds with interesting performances. Among natural polymers, the plant-based or microbial-based nanofibrillated cellulose (NFC) (Cheng et al., 2019b) has been frequently combined with the water/acid soluble polysaccharides (e.g., alginate (Markstedt et al., 2015), carboxymethyl cellulose (CMC) (Mohan et al., 2020), chitosan (Kamdem Tamo et al., 2021), etc.), or proteins (e.g., collagen/gelatin, silk (Filippi et al., 2020).) to prepared 3D scaffolds, suitable for long-term TE applications.

NFC exhibits high surface area, size (fibril width: 5–20 nm) and good biocompatibility and mechanical properties (Prakash Menon et al., 2017). It can also be used to support cellular processes during cell culturing and mimic the physiological functions of collagen, the main structural component of extracellular matrix (ECM) (Kummala et al., 2020). Recently, we used a combination of NFC and CMC for DIW (Mohan et al., 2020). CMC, a derivative of the natural polymer cellulose, is inexpensive, biocompatible, water soluble, has pH-dependent solubility and charge, and can be easily cross-linked/modified with various functional molecules (Gürer et al., 2021). It is structurally similar to NFC and exhibits a natural affinity to NFC through interfacial adhesion. Therefore, the addition of NFC to CMC can not only improve the ink flow properties (shear thinning) but also the flexibility, mechanical strength, and shape fidelity of the scaffold structure compared to other polysaccharides such as alginate, hyaluronic acid, etc (Mohan et al., 2018; Oliveira and Reis, 2011).

However, for most of the TE applications, the NFC-based scaffolds still show inadequate surface properties, such as interconnected pores and long-term dimensional and mechanical strength in a complex biological environment (e.g., cell growth medium) and under physiological conditions (pH 7.4 and 37°C). In such cases, the mentioned scaffolds’ properties can be improved by solution-based chemical (e.g., glutaraldehyde) (Jeon et al., 2019) or physical (e.g., Ca^2+^) cross-linking methods (Markstedt et al., 2015). These stabilization procedures often involve extensive modification steps or pretreatments with reactive chemical groups, which involve cytotoxic chemicals, demanding extensive purification (Petta et al., 2018). In this study, we want to address some of the drawbacks of NFC-based scaffolds described earlier. In particular, our motivation was to prepare 3D printed and freeze-dried NFC/CMC scaffolds cross-linked with citric acid (CA), a nontoxic and organic acid cross-linker. Cross-linking was achieved by dehydrothermal (DHT) heat treatment in the dry state. This combined novel approach was anticipated to provide NFC/CMC scaffolds with adequate interconnected pores/porosity, biocompatibility, and dimensional and mechanical stability in both dry and hydrated states. All these properties are required for long-term and successful cell growth. DHT-treatment is a solvent-free method and is frequently used to improve the mechanical properties of collagen-based biomaterials (Chen et al., 2020; Drexler and Powell, 2011). Although there are reports on the use of CA for cross-linking cellulose-based materials (Lungu et al., 2021; Quellmalz and Mihranyan, 2015), to our knowledge, no studies have been reported on the cross-linking of NFC/CMC scaffolds with CA by DHT-treatment and their use for the growth of human bone tissue derived osteoblast cells (hFOB).

In this study, we investigated for the first time the fabrication of mechanically stronger and porous NFC/CMC scaffolds by combining DIW 3D printing, freeze-drying, and DHT-assisted chemical treatment. 3D printed and freeze-dried NFC/CMC scaffolds were cross-linked with increasing concentrations of CA at elevated temperatures. We examined the influence of citric-acid-induced cross-linking density and its influence on scaffolds performance. Therefore, the neutralized scaffolds were analyzed concerning their morphology, composition, thermal behavior, swelling capacity, degradation, and mechanical strength in both dry and hydrated states. The applicability of the cross-linked scaffolds in TE was evaluated using the viability and proliferation of hFOB as a model system.

## Results and discussion

### Ink preparation and characterization

**Figure 1:**
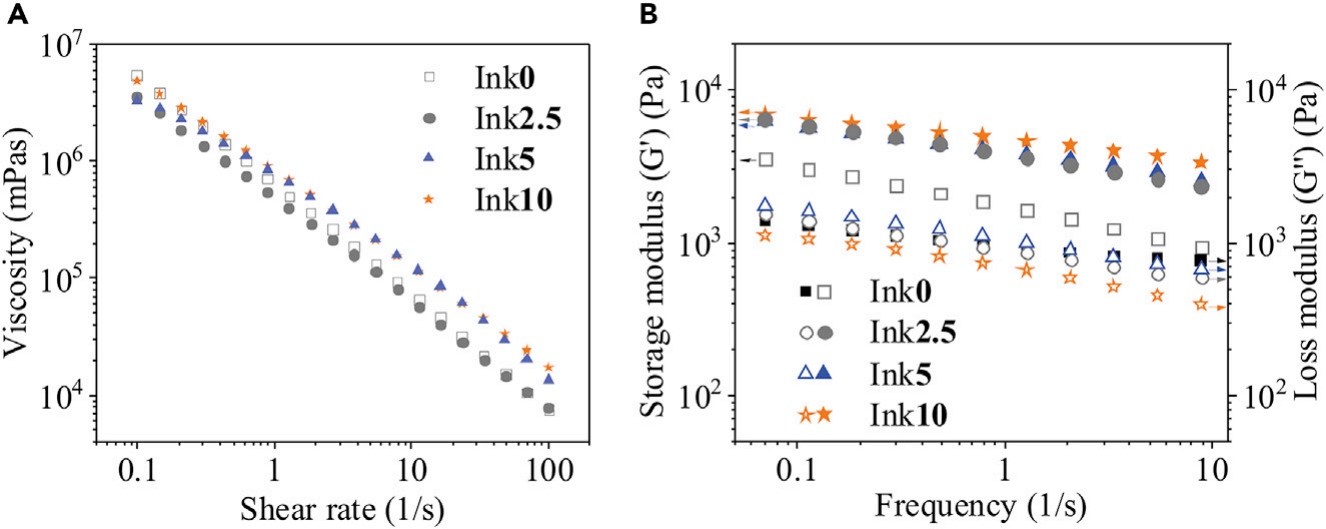
Rheological properties of the inks prepared with different amounts of citric acid concentrations (0 –10 wt.%) Viscosity curves (A) and frequency-dependency of the storage (symbols completely filled) and loss shear moduli (symbols not filled) (B) of the inks composed of nanofibrillated cellulose (NFC), carboxymethyl cellulose (CMC), and citric acid at various concentrations (0–10 wt.%).

In our previous work, we reported the preparation of bicomponent inks with constant solid content of NFC (1.5 wt.%) and CMC (6 wt.%). This ink was used for the fabrication of NFC/CMC scaffolds using DIW 3D printing (Mohan et al., 2020). To extend the applicability of our ink system and improve the mechanical properties of the scaffolds, we added citric acid (CA: 0 - 10 wt.%, see see Table 1) as a green cross-linker, in addition to NFC and CMC in the ink (see Table 1**)**. Figure 1 shows the viscosity and storage modulus of the NFC/CMC inks prepared with different concentrations CA (0–10 wt.%). As shown in Figure 1A, all investigated fluids featured very strong shear thinning behavior because of the intrinsic interfacial adhesion between NFC and CMC, favoring the rheological behavior and ensuring excellent printability of the inks (Mohan et al., 2020). In general, the influence of CA amount on viscosity is rather minor and expected for a small organic compound with high-water solubility.

**Table 1:** Preparation of inks based on nanofibrillated cellulose (NFC), carboxymethyl cellulose (CMC), and citric acid (CA) and their final compositions

Inks	NFC (g)	CMC (g)	CA (g)	Final solid content of each component in the ink		
				NFC (g)	CMC (g)	CA (g)
Ink**0**	50	6	0	1.5	6	0
Ink**2.5**	50	6	2.5	1.5	6	2.5
Ink**5**	50	6	5	1.5	6	5
Ink**10**	50	6	10	1.5	6	10

Bolded values correspond to the citric acid concentrations in the ink.

As shown in Figure 1B, all investigated inks behave similar to a rheological gel or a soft solid (the storage modulus G′ is higher than the loss modulus G″). Interestingly, the storage moduli are indeed affected by CA concentration and were generally increased by the addition of CA. The effect is even more obvious in the loss factor plot (Figure S1), which clearly shows an increase in elastic gel strength for higher amounts of CA and also a higher stability of the gel network in the measured frequency range. The ink containing 10 wt.% CA features the lowest loss tangents and therefore has the most pronounced elastic behavior. The higher gel strength of the samples containing CA can be explained by stronger interfacial adhesion of CMC and NFC, resulting in adsorption of CMC onto the NFC surfaces (Mohan et al., 2020). Such sorption behavior has been reported in similar systems in the presence of electrolytes, such as CaCl_2_ (Junka et al., 2014), because of screening of the repulsive charge interactions of cellulose and CMC; and CA as an electrolyte could induce similar effects.

### Scaffold’s design, neutralization, and optimization

**Figure 2:**
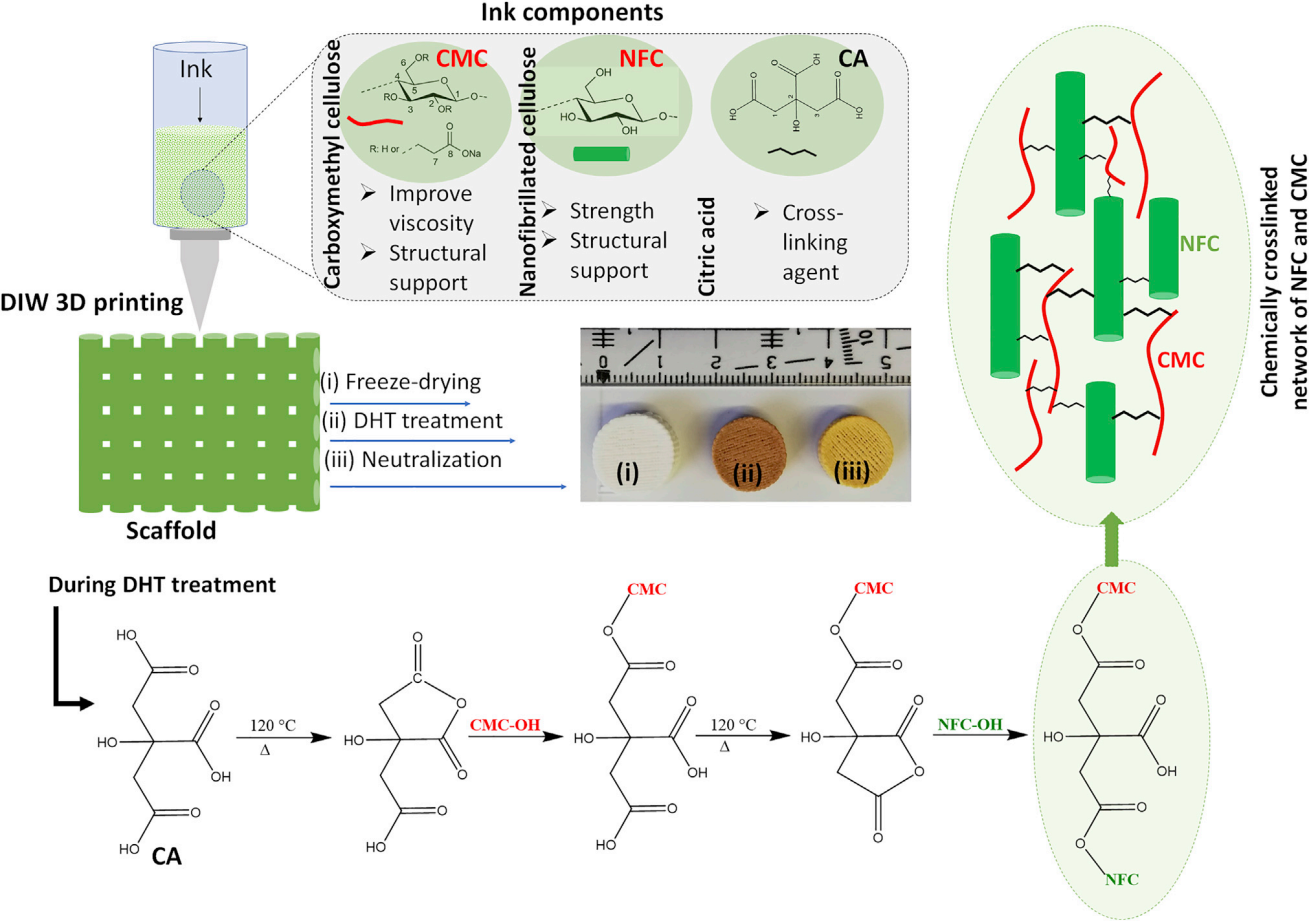
Illustration of the design of citric acid cross-linked three-dimensional scaffolds of nanofibrillated cellulose and carboxymethyl cellulose via the combination of direct ink writing printing, freeze-drying, and dehydrothermal heat treatment

To prepare CA-cross-linked NFC/CMC scaffolds with adequate pore size, mechanical properties, and dimensional stability in the hydrated state, which are demanded for long-term 3D cell culturing, the freeze-dried scaffolds were subjected to DHT treatment at an elevated temperature (120°C). The same treatment was done for CA-free scaffolds (Ink**0**/120°C). This solvent-free DHT treatment, performed in the dry state, was expected to result in the ester bond formation between the hydroxyl functional groups of NFC or CMC and carboxylic groups of CA in the scaffold. After DHT-treatment, the color of the CA-cross-linked scaffold was changed from white to yellow (see Figure S2), which is more pronounced the higher the concentrations of CA in the scaffold. This may be because of dehydration of CA and subsequent formation of unsaturated acids in the scaffold (see Figure 2) (Cai et al., 2019; Kučerová et al., 2016). The coloration of the scaffold can also be because of the degradation of NFC and CMC to a certain extent at higher temperature as reported in our previous work (Dobaj Štiglic et al., 2021). Our aim was to obtain acid-free scaffolds; otherwise, excess or residual acid could lead to undesirable cytotoxic effects. Therefore, all DHT-treated scaffolds cross-linked with different amounts of CA (2.5, 5, and 10 wt.%) were neutralized with NaOH (0.1 M). Although we performed the neutralization at different times (20, 40, and 60 min), the scaffolds were completely neutralized only at 60 min. This was verified by storing the scaffolds in biofluid under physiological conditions. The scaffolds neutralized for 20 and 40 min showed a change of color from pink to yellow, which was no longer the case after 60 min **(**Figure S3). This indicates that the remaining acids in the scaffolds were completely neutralized at this time. Therefore, we used 60 min as the neutralization time for the here-prepared CA-cross-linked scaffolds. All successfully cross-linked and neutralized scaffolds (Ink**2.5**/120°C/N, Ink**5**/120°C/N, and Ink**10**/120°C/N) were used for further characterization and biological evaluation. The non-cross-linked scaffold Ink**0**/120°C (without neutralization step) was used for comparison.

### Morphology and porosity

**Figure 3:**
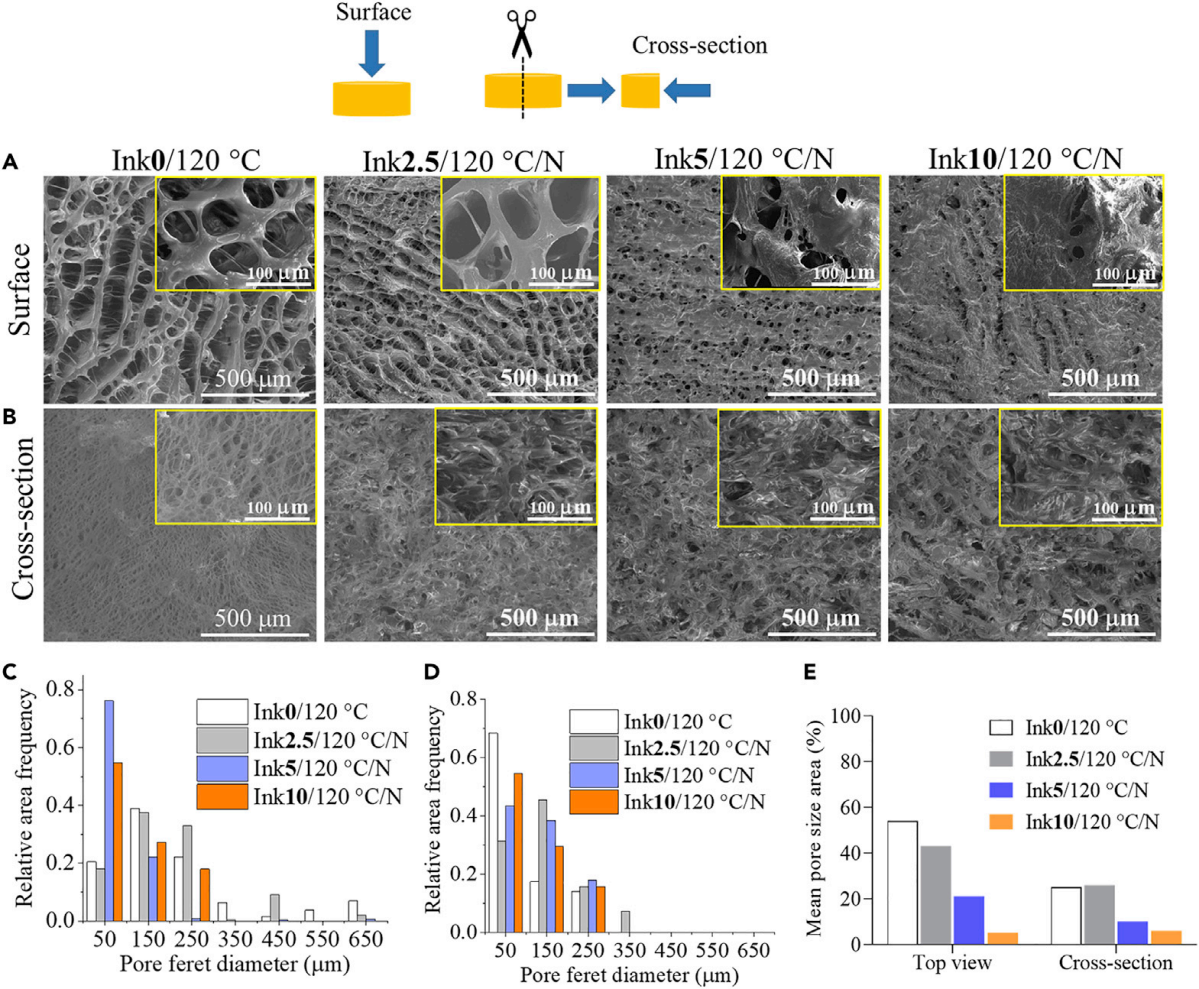
SEM morphology and pore size analysis (A) Top view (B) Cross-section (side view) of dry scaffolds cross-linked without (Ink0/120°C) and with different concentrations of citric acid (0 – 10 wt.%). Magnification in images is 100× and 1000x (insert). Mean Feret diameter (μm, C: surface and D: cross-section) and pore size area (%, E) of non-cross-linked and CA-cross-linked scaffolds.

The scanning electron microscopy (SEM) images of non-cross-linked and CA-cross-linked scaffolds are shown in Figure 3. An open porous morphology and interconnected pores can be seen on the surface and in the cross section of the non-cross-linked scaffold Ink**0**/120°C (CA-free, Figures 3A and 3B). Pore sizes of the samples were measured through analyses of the respective SEM images at the surface and cross section of the sample and ranged from 50 to 650 μm, whereas the observed pores at the surface were generally larger than those in the cross section of the samples (see Figures 3C and 3D). Compared to the non-cross-linked sample, Ink**2.5**/120°C/N showed higher surface pore size, ranging from 250 μm to 550 μm. Interestingly, both scaffolds: Ink**0**/120°C and Ink**2.5**/120°C/N showed a channel-like porous morphology. This kind of open, interconnected, and continuous porous structure and pores (size: 100–450 μm) are beneficial for attachment, migration and proliferation of cells, and effective nutrient transport, thus making them an attractive scaffold for soft and hard TE applications (Cheng et al., 2019a; Morejón et al., 2019). The other two CA-cross-linked scaffolds (Ink**5**/120°C/N and Ink**10**/120°C/N) exhibited different (closed) surface morphology and reduced pore sizes. Ink**5**/120°C/N showed more uniform small pores (50 μm) on the surface (see also Figure 3C) and larger pores (between 100 and 350 μm) in the cross section (Figure 3D). Ink**10/**120°C/N showed an even more closed surface morphology and fewer interconnected pores. It is evident that the Ink**5**/120°C/N and Ink**10**/120°C/N scaffolds cross-linked with higher concentrations of CA displayed smaller pores and less interconnected pores. Thus, these two scaffolds are less attractive for TE applications.

In general, there is a clear trend in the change of (open to closed) morphology and reduction of pore size as the concentration of the cross-linker CA increases (see Figure 3E). It has already been shown that the pore size of the sample depends on the size of the ice crystals formed during freeze-drying, which can be influenced by the freezing temperature rate, the chamber pressure, and the type of mold used. Furthermore, other factors such as the type of polymers, viscosity, solid content, and cross-linking type can also be vital in determining the pore size of resulting scaffolds (Li et al., 2017; Mirtaghavi et al., 2020). For example, the increased addition of large molecules, such as chitosan (Dobaj Štiglic et al., 2021; Lin and Yeh, 2012), polyvinyl alcohol (Kanimozhi et al., 2018), collagen (Pawelec et al., 2014; Pottathara et al., 2021), alginate (Bahrami et al., 2019), or smaller molecules e.g., citric acid (Wu et al., 2019a) and ferulic acid (Mathew and Abraham, 2008), have been shown to reduce the scaffold porosity/pore size. A similar behavior is also observed for our scaffolds and the increasing amount of CA. One explanation could be that the higher amounts of CA favor the formation of smaller ice crystals and thus smaller pore sizes in the scaffolds (Li et al., 2017).

**Figure 4:**
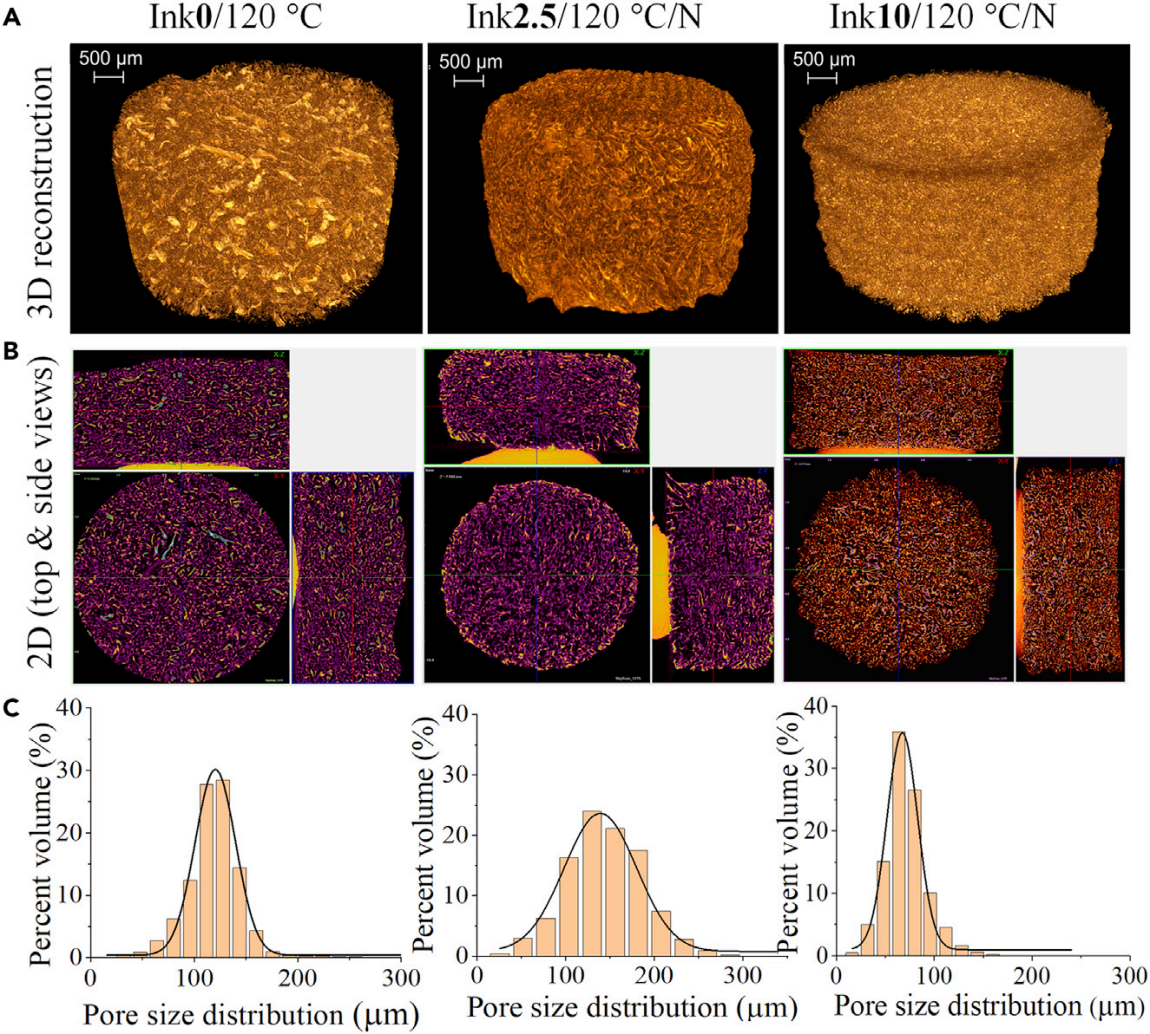
Micro-CT analysis of the dry scaffolds (A) 3D reconstruction and (B) 2D top and side views and (C) pore size distribution of scaffolds before and after cross-linking with different concentrations of citric acid.

Representative 2D and 3D micro-CT images of non-cross-linked (Ink**0**/120°C) and CA-cross-linked (Ink**2.5**/120°C/N and Ink**10**/120°C/N) scaffolds in the dry state are shown in Figure 4. We also determined the main structural parameters (open porosity and closed porosity volume in %) from 3D analysis and the results are shown in Table 2. This analysis was performed to study the pore morphology and spatial heterogeneity and to quantify the porosity of the scaffold. The reconstructed 3D image (and Video S1) of Ink**0**/120°C (Figure 4A, left) proves the highly porous morphology and internal micro-architecture. Similar features are also observed for Ink**2.5**/120°C/N (Figure 4A, middle, and Video S2). However, these features are less pronounced for Ink**10**/120°C/N (Figure 4A, right, and Video S3), the scaffold cross-linked with the highest concentration of CA (10 wt.%). The representative 2D images (top and side views, Figure 4B, see also Figures S4–S6) show the structure of the NFC/CMC solid fraction and provide information about the distribution of interconnected pores/structure. The pore size distribution profile (Figure 4C) shows that the pore sizes range between 50 and 180 μm (10-20%), 50 and 280 μm (10-25%), and 50 and 100 μm (5-30%) for Ink**0**/120°C, Ink**2.5**/120°C/N, and Ink**10**/120°C/N, respectively. The calculated pore wall thickness in CA-cross-linked/non-cross-linked scaffold is in the range of 35–38 μm (Table 2). Even though the pore sizes of the scaffolds decreased with increasing concentration of CA as demonstrated by both SEM and micro-CT, the latter showed lower values of pore size compared to SEM. The micro-CT 3D pore size evaluation is based on a sphere fitting algorithm. It is the most precise parameter considering the orientation-dependent direct 3D analysis, whole specimen evaluation, irregular pore assessment, low image processing bias, and lack of subjectivity in the assessment (Bartoš et al., 2018; Duarte et al., 2020). On the other hand, SEM analysis is based on mechanical sectioning and special treatment of the specimen and is limited to 2D structure and number of sections. In addition, the resolution of the micro-CT is lower compared to SEM, which reduces the detection of fine pore intersections (Wu et al., 2019b). These factors may result in structural alterations and complicate the accurate assessment of both the pore margin and the interconnection (Bartoš et al., 2018). When compared with the literature values reported for various natural (e.g., chitosan) or synthetic polymeric (e.g., polycaprolactone) scaffolds for TE purposes, the pore sizes observed for Ink**2.5**/120°C/N are suitable for cell growth applications (Cheng et al., 2019a). The non-cross-linked Ink**0**/120°C showed open or total pores (porosity) value of 86.3%, which is defined as spaces within the scaffold structure that displayed a connection with the surrounding space outside the object. This porosity value is slightly lower for CA-cross-linked samples (Ink**2.5**/120°C/N:77.7% and Ink **10**/120°C/N:74.4%). Closed pores/porosity values (sum of scaffold volume and the volume of closed pores) ranged from 0.0002 to 0.003%, which is defined as spaces surrounded by the scaffold structure with no connection in 3D with the surface of the scaffold. The object volume is taken to mean the volume of the scaffold material without the pores. The observed very low degree of closed porosity indicates a high degree of interconnected pores within the scaffold in the dry state (Suchý et al., 2018).

**Table 2:** Results of the statistical analysis of micro-CT data obtained from dry and wet scaffolds

Samples	Porosity (%)		Object volume (%)	Pore wall thickness (μm)
	Total/Open porosity	Closed porosity		
**In dry state**				

Ink**0**/120°C	86.3	0.003	13.7	38.5
Ink**2.5**/120°C/N	77.7	0.005	22.3	34.9
Ink**10**/120°C/N	74.4	0.01	25.6	35.3

**Ink 2.5/120°C/N in wet state**				

24 h	30.4	0.3	69.6	785.8
48 h	18.8	2.9	81.2	810.3
72 h	20.3	3.4	79.7	609.5

Bolded values correspond to the citric acid concentrations in the ink.

Video S1. Visualization of 3D reconstructed image of Ink0/120°C scaffold in dry state (related to Figure 4)

Video S2. Visualization of 3D reconstructed image of Ink2.5/120°C scaffold in dry state (related to Figure 4)

Video S3. Visualization of 3D reconstructed image of Ink10/120°C scaffold in dry state (related to Figure 4)

**Figure 5:**
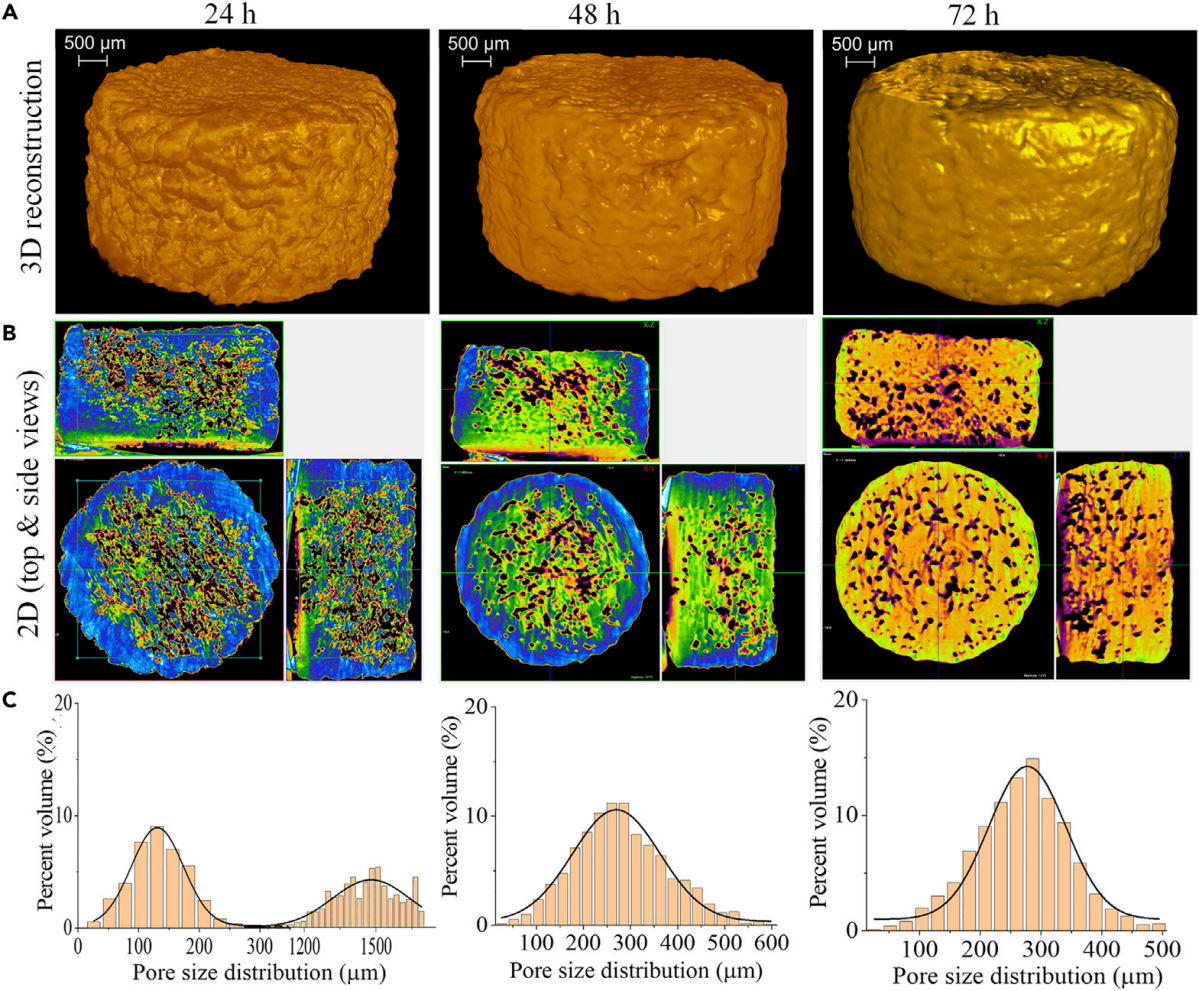
Micro-CT analysis of the wet scaffolds (A) 3D reconstruction and (B) 2D top and side views and (C) pore size distribution of scaffold Ink2.5/120°C/N stored in water at different time points.

Structural changes associated with hydration are important, but their assessment is difficult. We used micro-CT to analyze the morphological and structural features of the scaffold (Ink**2.5**/120°C/N) in the hydrated state. Before this experiment, the scaffold was completely immersed in water for 24, 48, and 72 h, respectively. The 2D and 3D reconstructed images and pore size distribution obtained from this measurement are shown in Figure 5. The scaffold Ink**2.5**/120°C/N was chosen for this analysis because it showed promising characteristics such as interconnected porous structure, pore sizes, and porosity (according to SEM/micro-CT results, Figures 3 and 4), suitable for soft or hard TE application (Cheng et al., 2019a). In general, change in pore structure and morphology of the Ink**2.5**/120°C/N scaffold immersed in water at different time intervals is clearly visible compared to the dry scaffold (0 h) because of hydration. These changes in structural features are more pronounced with longer hydration time. The total or open porosity of the dry scaffold is reduced to 56% after 24 h (see also Video S4). A further 10% reduction in pore size is observed after 48 (Video S5) and 72 h (Video S6) of equilibration. The closed porosity is significantly increased (60–680%) in hydrated scaffolds compared to dry scaffolds (see Table 2). Interestingly, the scaffolds hydrated for 24 h showed two types of pore size distributions: micro pore size (80–180 μm) and macro pore size (1200–1500 μm). At 48 and 72 h, the observed pore size distribution is in the range between 40 and 450 μm. Despite the reduction in porosity, the hydrated scaffolds showed increased pore size than the dry scaffolds. It is also evident that the pore size increased 2-fold for the scaffolds that were hydrated for longer times (48–72 h). This increased wet pore size is important for cell adhesion and tissue growth (Suchý et al., 2018). The hydrated scaffold is close to the regeneration of cartilage (e.g., ear) and bone (e.g., cortical) tissue in terms of pore size (porosity: 10–30% and pore size: 50–500 μm) (Abbasi et al., 2020). Thus, from the perspective of structural parameters in the hydrated state, the scaffolds have the potential to be used as a biotemplate/bioscaffold for TE applications. It should be mentioned that scanning of hydrated scaffolds presents technical difficulties, e.g., insufficient X-ray contrast between the scaffold matrix and the solution or motion artifacts caused by gravitation, specimen rotation, and swelling. In such cases, the X-ray contrast must be enhanced by contrast agents (e.g., iodine solution, calcium thiocyanate, and phosphotungstic acid). However, their influence on the scaffold properties is still relatively unknown and may therefore lead to alterations.

Video S4. Visualization of 3D reconstructed image of Ink2.5/120°C scaffold after immersing in water for 24 h (related to Figure 5)

Video S5. Visualization of 3D reconstructed image of Ink2.5/120°C scaffold after immersing in water for 48 h (related to Figure 5)

Video S6. Visualization of 3D reconstructed image of Ink2.5/120°C scaffold after immersing in water for 72 h (related to Figure 5)

### Structure and thermal properties

**Figure 6:**
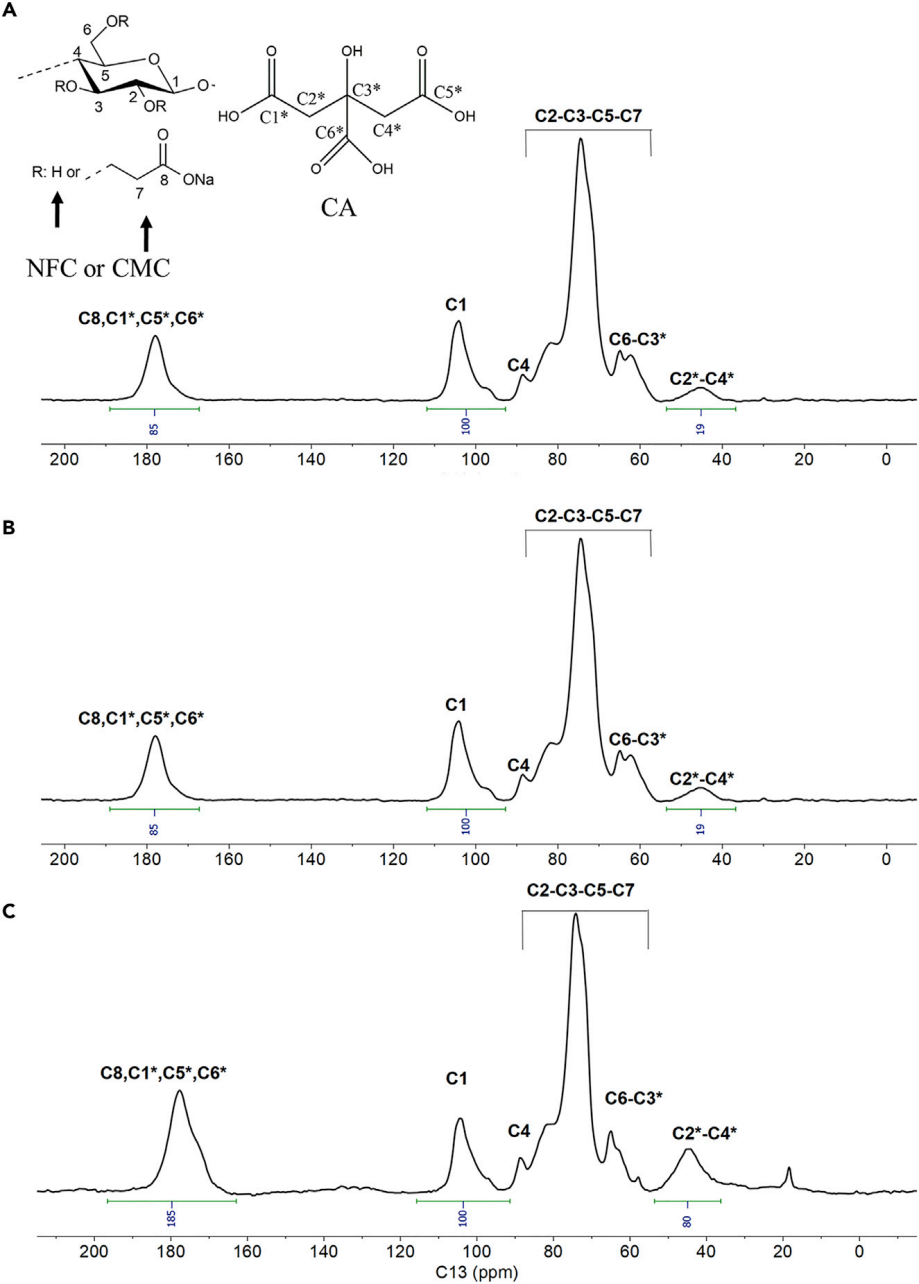
^13^C-solid state NMR spectra of NFC/CMC scaffolds cross-linked with different amounts of citric acid (–2.5–10 wt.%) (A) Ink2.5/120°C/N. (B) Ink5/120°C/N. (C) Ink10/120°C/N.

Figure 6 shows ^13^C NMR spectra of NFC/CMC scaffolds cross-linked with three different CA concentrations (2.5, 5, and 10 wt.%). The Ink**0**/120°C (Figure S7B) showed the characteristic peaks because of the carbon atoms of NFC or CMC appeared at *δ*_C_ 61 (C6), 70–80 (C2, C3, C5, and C7), 81 (C4), and 105 ppm (C1; the respective carbons are numbered in Figure 6). Besides this, the carboxyl carbon (C8) of the CMC carboxylate group is observed at *δ*_C_ 178 ppm (Dobaj Štiglic et al., 2021; Gürer et al., 2021; Mohan et al., 2020) (see Figure S7). All these characteristic peaks are also observed for CA cross-linked sample (Ink**2.5**/120°C/N, Figure 6A), in addition to the methylene carbon atoms of CA at *δ*_C_ 45 ppm (C2∗-C4∗) (Sun et al., 2019). Besides, the carboxyl carbon (C=O) at *δ*_C_ 178 ppm showed broadening, which could be because of the presence of free acid (-COOH) and ester cross-links between NFC or CMC and CA within the scaffolds. With increasing CA concentrations in the scaffold (Ink**5**/120°C/N and Ink**10**/120°C/N, Figures 6B and 6C), the intensity and broadening of the signals attributed to the carboxyl carbon and methylene carbon atoms of CA are also increased. This may be attributed to the increased formation of ester bonds between CA and NFC and/or CMC polymer. To quantify the degree of cross-linking, we used relative integral values that are reported with respect to the integral region of C1 carbon atoms of NFC/CMC between *δ*_C_ 97-105 ppm, which was arbitrarily set to 100. The other two areas used for comparison belong to the methylene carbon atoms of CA at *δ*_C_ 36-53 ppm and the C=O carbon atoms at *δ*_C_ 167-189 ppm. Furthermore, the integral values ratio between carboxyl and methylene regions was calculated for each sample (Table 3). A lower ratio value indicates that there are fewer protons close to the carboxyl C=O carbon, which promotes the formation of additional ester bonds as the citric acid concentration increases.

**Table 3:** Integral value ratio between carboxyl and methylene regions

Samples	Integral value ratio
Ink**2.5**/120°C/N	4.5
Ink**5**/120°C/N	3.5
Ink**10**/120°C/N	2.3

Bolded values correspond to the citric acid concentrations in the ink.

**Figure 7:**
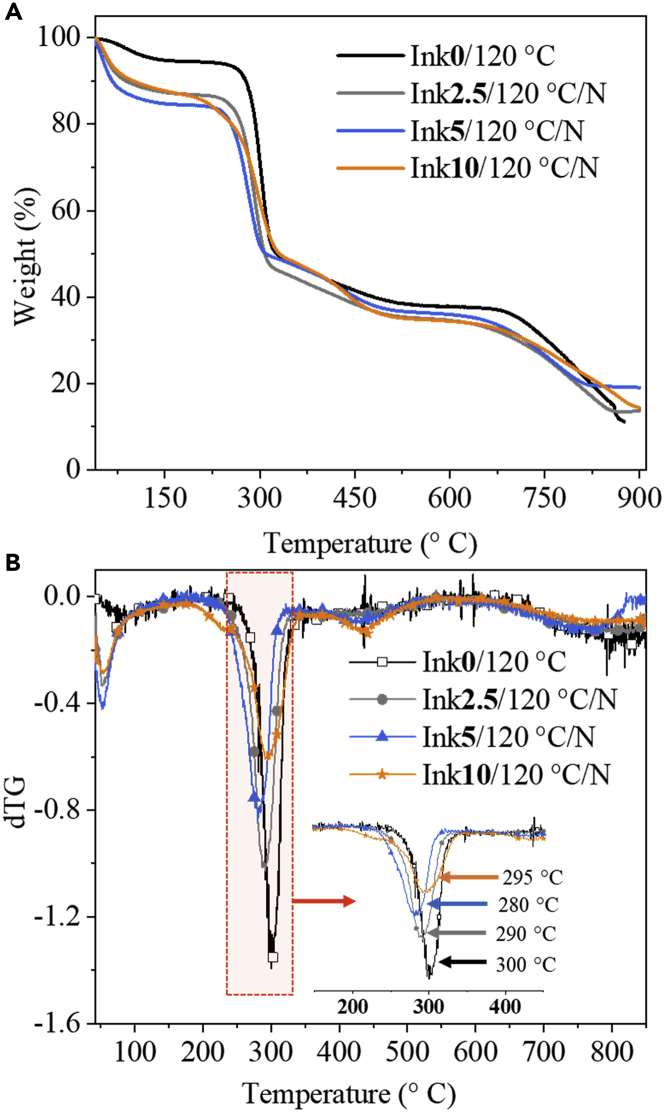
Thermal stabilities of cross-linked and non-cross-linked scaffolds TGA (A) and dTG (B) curves of NFC/CMC scaffolds cross-linked with different citric acid concentrations.

We also performed thermogravimetric analysis (TGA) to determine the thermal stability of the scaffolds. The TGA curves and their derivatives (dTG, the rate of mass loss) of non-cross-linked and cross-linked samples with CA are shown in Figure 7. The observed thermal behavior for the neat polymers is depicted in Figure S8. In general, the thermal stability of CA-cross-linked/non-cross-linked NFC/CMC scaffolds changed considerably compared to neat NFC and CMC, and degradation occurs in two steps. Otherwise, the observed decomposition patterns for CA-cross-linked and non-cross-linked scaffolds are like that of neat polymers. The observed peaks in dTG, between 50°C and 100°C (first step) for the neat NFC and CMC, did not appear for any of the NFC/CMC scaffolds. This can be associated with the removal of physically bound water molecules. In the second step of degradation (between 250°C and 350°C) the DTG curves in Figure 7B (see insert) indicate a maximum weight loss rate of 35% for NFC/CMC composite scaffolds compared to the neat polymer (NFC: 64% and CMC: 25%). This is because of CMC or CA decarboxylation reactions and the onset decomposition of NFC among other factors (Dobaj Štiglic et al., 2021; Mohan et al., 2020). Surprisingly, cross-linking with CA at all concentrations does not considerably influence the thermal stability of the scaffolds compared to non-cross-linked ones (Ink**0**/120°C) despite the large differences in the mechanical properties of the scaffolds as a function of CA concentration (see below).

### Wettability, swelling, and weight loss

Because the wettability of scaffolds can affect cell adhesion behavior, we performed static water contact angle (SCA(H_2_O)) measurements for both non-cross-linked and cross-linked samples. However, it was not possible to determine the (SCA(H_2_O)) values for all samples. The water droplet was rapidly absorbed (within a few seconds, see Figure S9) as soon as it touched the scaffold surface. This indicates that all scaffolds are very hydrophilic, which is beneficial for the rapid uptake and transport of nutrients in the process of cell and tissue growths.

**Figure 8:**
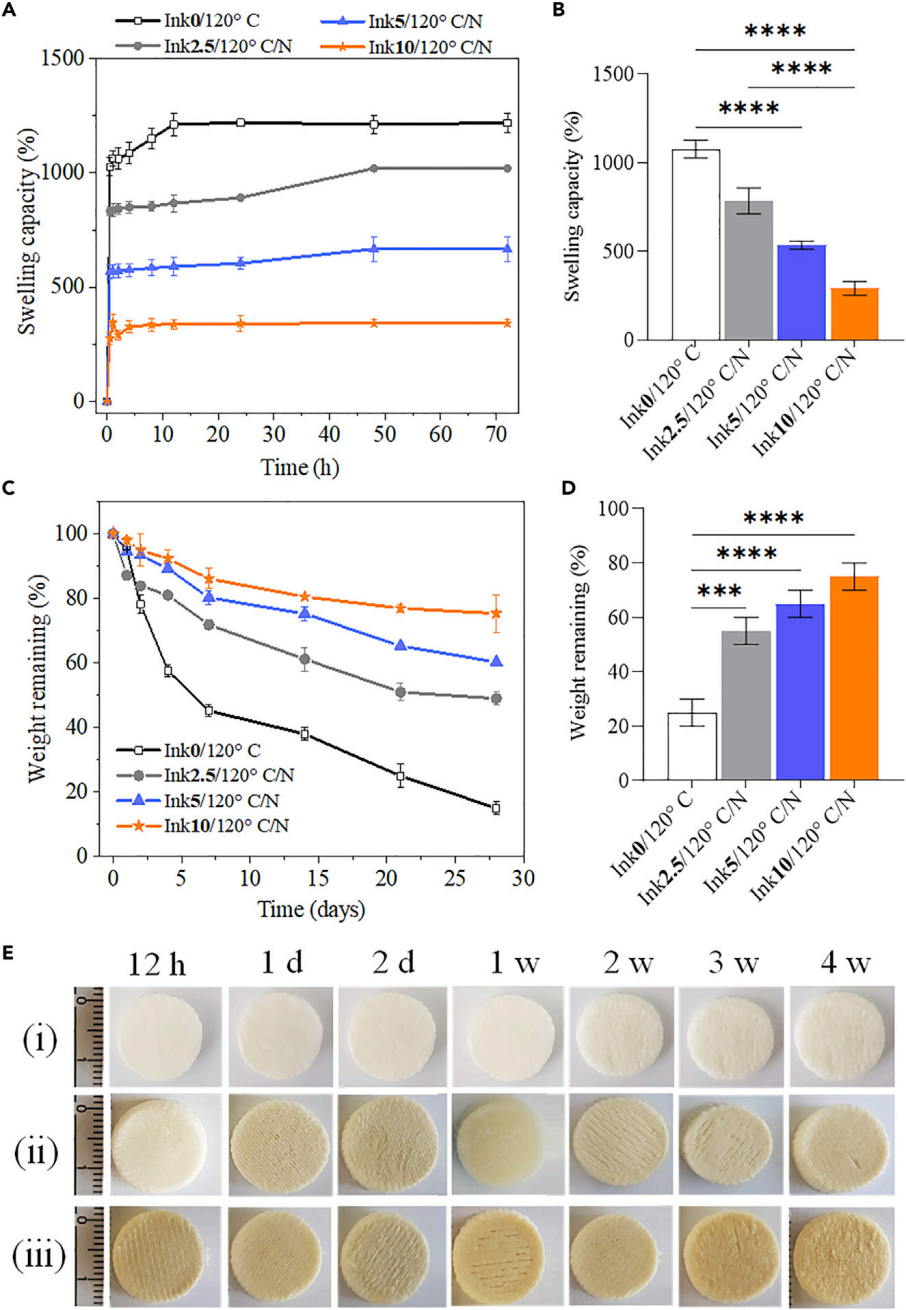
Swelling and degradation of cross-linked and non-cross-linked scaffolds Swelling (A, B) and weight loss (C, D) of NFC/CMC scaffolds cross-linked with different citric acid concentrations. (E) Images of NFC/CMC scaffolds (i: Ink**0**/120°C, ii: Ink**2.5**/120°C/N and iii: Ink1**0**/120°C/N) taken after the weight loss test at different times. Data analysis was done by one-ANOVA with the Dunnett test, and values are presented as ± SD; ∗∗∗p < 0.05, ∗∗∗∗p < 0.05 (compared to control Ink**0**/120°C).

Swelling is a key parameter to determine how the scaffolds respond to contact with biological medium (e.g., cell culture medium) and affect cell adhesion behavior. Percent swelling of scaffolds cross-linked without and with CA at different concentrations is shown in Figures 8A and 8B. The swelling of the non-cross-linked and porous scaffold Ink**0**/120°C increased rapidly to 900% within 30 min and reached 1200% saturation in the next few hours. It has been reported that hydrogels prepared with high cross-linking densities showed decreased swelling behavior (Grabska-Zielińska et al., 2020; Lin and Yeh, 2012; Wang et al., 2013). Likewise, our CA-cross-linked scaffolds showed reduced biofluid uptake capacity at higher cross-linking densities (Figures 8A and 8B). The Ink**2.5**/120°C/N scaffold decreased swelling to 800% in 30 min and reached a saturation of 1000% during the next few hours. Similarly, the swelling percentage gradually decreased for the other two concentrations of CA. The total biofluid uptake capacity of the scaffolds decreased in the following order: Ink**0**/120°C (1150 ± 80 g/g) < Ink**2.5/**120°C/N (890 ± 75 g/g) < Ink**5**/120°C (600 ± 40 g/g) <Ink**10**/120°C (325 ± 25 g/g). It is also clear that the fluid uptake property of the scaffold decreases with decreasing porosity as demonstrated by SEM and micro-CT results (Figures 3 and 4). The lower biofluid uptake capacity of the CA-cross-linked scaffolds compared to non-cross-linked Ink**0**/120°C scaffolds indicates the occurrence of cross-linking reactions. This is in good agreement with the NMR results, which showed increased ester bond formation or cross-linking with increasing CA concentration. It is suggested that DHT-assisted CA cross-linking formed a stronger bonding and tighter cross-linked network structure by consuming hydrophilic functional groups (hydroxyl and carboxyl groups) in NFC and CMC, allowing less water to enter the scaffolds and thus reducing swelling. Similar results were reported for other cross-linked scaffolds/hydrogels, such as hyaluronic acid/gelatin/collagen, chitosan/collagen, carboxymethyl cellulose/poly(ethylene oxide) and chitosan/collagen/silk fibroin, which were cross-linked with carbodiimide (Wang et al., 2013), citric acid (Kanafi et al., 2019), genipin (Lin and Yeh, 2012), and dialdehyde starch (Grabska-Zielińska et al., 2020), etc. The observed high fluid holding capacity of the scaffold is favorable for cell adhesion and growth and enables efficient transport of nutrients from the scaffold to cells in the culture system.

3D Scaffolds for TE applications not only need to be mechanically stable to support cell adhesion and proliferation without collapsing, but they also need to be gradually replaced by native ECM and therefore (slowly) biodegradable (Dobaj Štiglic et al., 2021; Milojević et al., 2019; Mohan et al., 2018; Reddy et al., 2021). Therefore, we investigated the *in vitro* degradation of scaffolds in biofluids at 37°C and pH 7.4, mimicking the native tissue environment, and the results are shown in Figures 8C and 8D. A clear difference in the degradation profiles is observed for CA-cross-linked and non-cross-linked samples. Over a period of 4 weeks, the non-cross-linked Ink**0**/120°C showed a significant weight loss (75%) compared to the CA-cross-linked ones (25-50%). Although the significant differences in weight loss are observed between the cross-linked samples, the weight loss of the cross-linked samples decreased linearly with increasing CA concentration (Figure 8D). The observed weight loss is in the following order after 4 weeks of *in vitro* degradation: Ink**0**/120°C (75 ± 5%) < Ink**2.5/**120°C/N 50 ± 6%) < Ink**5**/120°C (40 ± 5%) <Ink**10**/120°C (25 ± 4%). Figure 8E shows the images of *in vitro* degraded cross-linked scaffolds over a period of 4 weeks. No significant change in shape or structural collapse is observed for cross-linked scaffolds compared to the non-cross-linked ones. It is suggested that the ester cross-linking achieved by the DHT-aided treatment improves the long-term dimensional and structural stability of the scaffold in biofluid under physiological conditions. The chemically cross-linked NFC/CMC scaffolds with such versatile functional properties (e.g., stability, charges, etc.) obtained by a solvent-free process have high potential for use in long-term *in vitro* or even *in vivo* experiments.

### Mechanical analysis

**Figure 9:**
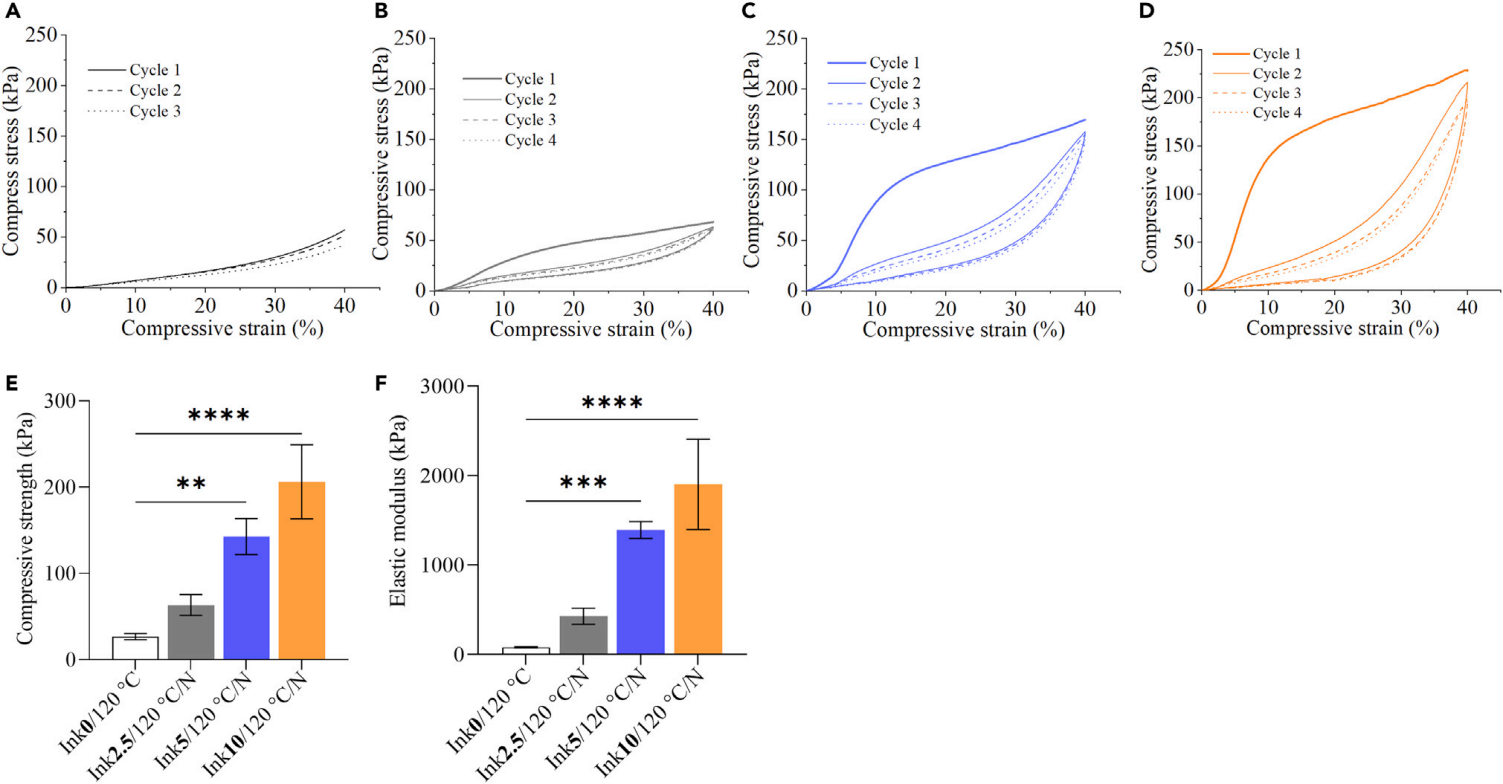
Cyclic compression curves of the of NFC/CMC scaffolds cross-linked with different CA concentrations (A: Ink**0**/120°C, B: Ink**2.5**/120°C/N, C: Ink**5**/120°C/N, D: Ink**10**/120°C/N). Compressive strength (at 30% strain) (E) and elastic modulus (F) were extracted from compression tests in Figures 9A–9D. Data analysis was done by one-ANOVA with the Dunnett test, values are presented as ± SD; ∗∗p < 0.05, ∗∗∗∗p < 0.05 (compared to control Ink**0**/120°C).

The mechanical properties of the (hydrated) scaffolds were analyzed after DHT and equilibration in biofluid by unconfined compression testing as shown in Figure 9. As expected, the mechanical properties increased with increasing amount of cross-linker, which is well demonstrated in the compression tests of the samples (Figures 9B–9D) as well as in Figures 9E and 9F, comparing the compressive strength (compressive stress at 30% strain) of the samples. In comparison to the non-cross-linked Ink**0**/120°C, addition of 10 wt.% CA increased the compressive strength almost 8 times from 27 kPa to 206 kPa. The effect is even further pronounced in the elastic modulus (Figure 9F), which is elevated by more than one magnitude from 79 kPa to 1.9 MPa. The same trend is also observed in dynamic compression measurements in a dynamic mechanical analysis tester, although the differences are less pronounced than in the static measurements (see Figure S8). CA clearly increased the elastic moduli of the scaffolds (Figure 9F) and decreased damping factor, further demonstrating stronger elasticity of the CA-cross-linked samples. Based on these results, we performed cyclic compression tests for cross-linked samples (Figures 9A–9D), and it can be stated that all CA-cross-linked samples recover well the dimensions upon compression to 40% strain. This is well demonstrated by the relaxation curves. For the non-cross-linked sample Ink**0**/120°C (CA-free), we did not measure the cyclic compression test because of its poor elastic properties as illustrated in unconfined compression tests (Figure 9A) and dynamic mechanical analyses (Figure S10). As shown in Figures 9B–9D for CA-cross-linked samples, similar compressive stress values at maximum strain are obtained for all cycles of one respective scaffold. However, it is obvious that the compression curve shape drastically changed upon the initial first cycle, and the elastic response (expressed in the initial slope of the test) is strongly reduced. Based on the design of the scaffolds, we suspect that this is because of partially broken interactions between individual contacting printed strands, probably occurring above the yield strength at approx. 8% strain. This did not influence the shape and general dimension stability but decreased the elastic behavior of the scaffold and could be further improved by varying the print architecture in follow-up studies. In general, we observed a significantly increased mechanical strength and elasticity of the samples upon chemical cross-linking with CA via DHT treatment. This further amplified the effect of initial physical cross-linking between NFC and CMC (Mohan et al., 2020). Based on the inhibiting effect of water in the chemical dehydration reaction of the initially wet system, we believe that water removal during DHT first ensures good physical interaction/cross-linking of NFC and CMC, which is then at a later stage chemically set through esterification reactions between the matrix components with CA. The mechanical properties, for example, elastic modulus, of our CA-cross-linked hydrated scaffolds are higher/comparable to that of reported biopolymers, such as alginate (10-36 kPa) (Hyland et al., 2011), chitosan/agarose (40 kPa) (Felfel et al., 2019), methyl cellulose/methacrylate (8-18 kPa) (Stalling et al., 2009), oxidized dextran/gelatin (0.5–1 kPa) (Berillo and Volkova, 2014), silk fibroin/hydroxyapatite (7-14 kPa), chitosan/chondroitin sulfate (18 kPa) (Yang et al., 2011), and the native cartilage (1–20 MPa). However, the bone meniscus matrix (10-12 MPa) exhibits higher values in the hydrated state. This can be matched with further modification of the scaffolds reported in this study. For example, the mechanical properties of the scaffolds could be increased by the additional incorporation of different bioactive components such as fibrous collagen or silk, elastin, etc.

### *In vitro* biocompatibility analysis

To confirm the potential of the developed materials for TE applications, we performed some preliminary cell testing. Because our future target is osteochondral interface related applications, it seemed appropriate to use human bone-derived osteoblasts for these tests. To this end, we performed three types of tests. First, an MTT assay was performed with the extracts of the samples incubated overnight to confirm the biocompatibility of potential sample degradation products. This was followed by two tests with the final scaffolds (first, the Live/Dead assay to prove the biocompatibility of the scaffolds, and then the quantitative XTT assay to measure cell viability after predefined time points).

**Figure 10:**
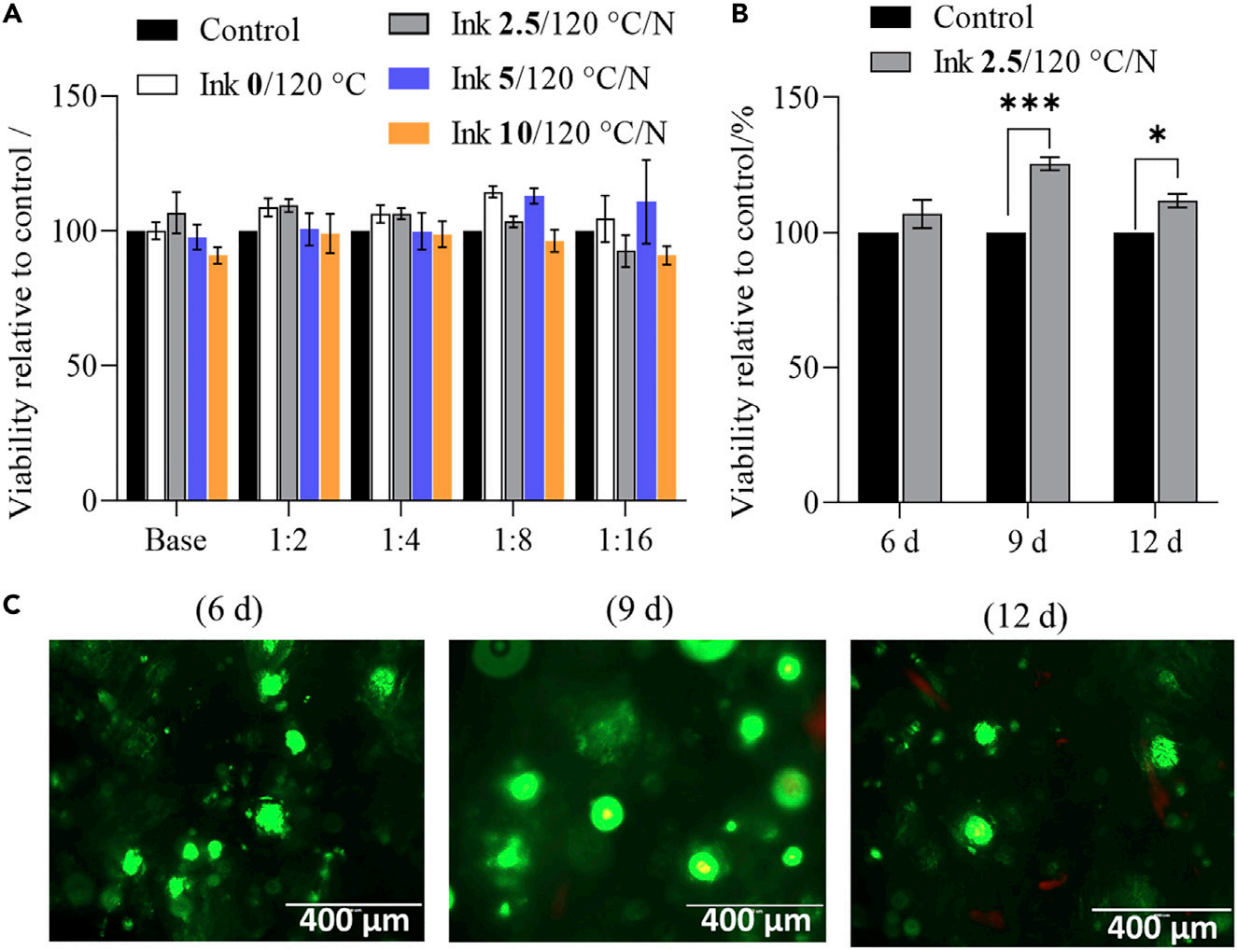
Human osteoblast cell’s viability exposed to the prepared samples (A)MTT assay on sample extracts at different dilutions; experiments were performed for 24 h. (B)XTT results one the scaffold Ink **2.5**/120°C/N tested at different time intervals. (C)fluorescence images of osteoblast cells cultured on scaffold Ink 2.5/120°C/N stained using the Live/dead assay after 6, 9ays and 12 days. Data analysis was done by one-student t-test (unpaired), values are presented as ± SD; ∗∗∗p < 0.05, ∗p < 0.05, (compared to control scaffold Ink **2.5**/120°C/N, tested without cells).

As mentioned earlier, the MTT assay test was first performed using the human bone tissue derived osteoblast cells (hFOBs) to evaluate the biocompatibility of the non-cross-linked and CA-cross-linked scaffolds (see Figure 10). Several conclusions can be drawn from the results. First, the percentage of viable cells is comparable (in some cases even higher) to the control for all tested scaffolds (Figure 10A). This means that the sample extracts are not toxic and that potential washouts or degradation products do not cause changes in pH nor local “overdosing” of components that could have influenced cell viability. Finally, in both undiluted and diluted forms, a significant increase in cell viability is observed for CA-cross-linked Ink**2.5**/120°C/N compared to the control sample (Advanced DMEM) and non-cross-linked sample. This implies that the cross-linked scaffolds have a significant effect on the viability of hFOBs. This effect is assumed to be because of changes in the surface morphology (i.e., open, cross-linked, and continuous porous structure) of the scaffolds rather than any biological effect that the citric acid may have had (Raucci et al., 2015). It could be that the scaffold’s porous morphology suits the osteoblasts cells, which in their native environment (i.e., the bone) attach most of the time to “solid substrates”.

Because the Ink**2.5**/120°C/N scaffold extracts showed improved viability and considering the desired morphological and structural features (i.e., higher porosity and continuously interconnected pores, see Figure 3A and Table 2), we selected this scaffold for the other two cell testing methods. Both tests (XTT and Live/dead assays) can be considered as “direct-contact” testing methods, because the cells are directly added and grown on the prepared scaffolds. Figure 10B shows the XTT assay results (XTT assay is similar to the MTT but can be applied directly to the scaffolds, because the final formazan dissolution step is omitted) obtained for Ink**2.5**/120°C/N at different time intervals. The results show a trend toward increased cell viability over time compared to the control sample. The control sample in this case was the “bare” Ink**2.5**/120°C/N sample but without added cells. Using it as a control, we can show that the difference in measured formazan formation is indeed because of the metabolically active cells on the scaffolds and not to possible coloration of the scaffolds with the dye. Long-term stable or even improved cell growth is the essence of successful scaffolds for TE applications (Collins et al., 2021; Zhang and King, 2020), which was confirmed using this assay.

In the final cell test, the scaffolds were investigated using the Live/Dead assay, which “visually” shows viable and dead cells on the scaffold’s surface, in this case at various time points. For this purpose, hFOBs were seeded directly on Ink**2.5**/120°C/N (as described in the Method details section), followed by staining with the Live/Dead kit, showing viable (green) and dead (red) cells. Figure 10C shows the fluorescence images of the osteoblast cells after 6, 9, and 12 days of incubation. The images show that the cells are observed in clustered form and are viable at most time points. After 12 days of exposure, some dead cells start to appear, which is normal for such long-term TE testing methods. Nevertheless, the majority of cells are growing, viable, and healthy.

Overall, the combined results of this work clearly show that CA-cross-linked scaffolds exhibit no toxicity to cells and allow long-term growth of human bone-derived cells. This proves that the scaffolds are biocompatible and are promising candidate materials for bone TE applications.

### Conclusions

In this study, we prepared and analyzed the polysaccharide-based porous 3D scaffolds from the ink composed of NFC, CMC, and CA at different concentrations. All inks—regardless of the CA concentration (0-10 wt.%)—exhibited a higher viscosity, shear thinning properties, and excellent shape fidelity of the freshly printed structures. The combination of DIW 3D printing, freeze-drying, and DHT-treatment resulted in a dimensionally and mechanically stable cross-linked scaffold. The heat-assisted latter treatment induced the ester bond formation or cross-linking between NFC or CMC and CA in the scaffold as confirmed by using solid state ^13^C NMR spectroscopy. The number of ester bond formation increased with increased concentrations of CA. The non-cross-linked (Ink**0**/120°C) and cross-linked scaffolds (Ink**2.5**/120°C/N) in dry state with lower CA concentration (2.5 wt.%) showed a highly porous morphology, increased porosity (74-86%), and interconnected pores (100-450 μm) compared to the scaffolds cross-linked with higher CA concentrations (5 and 10 wt.%) as demonstrated by scanning electron microscopy and micro-CT analysis. This indicates that the scaffold’s porosity/pore sizes decreased with increased cross-linking density as the concentration of CA increased, which is in good agreement with ^13^C NMR results. The hydrated scaffold of Ink**2.5**/120°C/N showed significantly reduced porosity and increased pore size than the dry scaffold. When compared to non-cross-linked scaffold (Ink**0**/120°C, without citric acid), the cross-linked one exhibited reduced swelling and degradation. This can be related to the reduced pore size as the concentrations of CA increased as demonstrated by SEM and micro-CT analysis. The mechanical properties of the cross-linked scaffolds in hydrated state were also changed as a function of CA concentration. Compared to non-cross-linked scaffolds, the mechanical properties (compressive strength: 2- to 3-fold and elastic modulus: 2- to 8-fold) of CA-cross-linked and hydrated scaffolds were increased significantly. Both non-cross-linked and cross-linked scaffolds were biocompatible, but the scaffold (Ink**2.5**/120°C/N) cross-linked with lower CA concentration was found to be suitable for long-term tissue growth. Considering the water-based and toxic solvent-free organic acid cross-linking method used for the fabrication 3D scaffolds from natural polysaccharide materials, it could be stated that our approach holds a huge potential not only in developing mechanically stronger but also long-term biocompatible scaffolds for various TE applications (e.g., bone).

### Limitations of the study

In this study, we used different concentrations (0–10 wt.%) of citric acid as a green cross-linker to control pore size in mechanical and dimensionally stable scaffolds. However, it turned out that the scaffolds cross-linked with 5 and 10 wt.% scaffold reduced the pore size (porosity and interconnected pores). Therefore, we did not perform any long-term biocompatibility and cell growth studies on these two types of scaffold. We also found out that the osteoblast cells did not adhere homogeneously and cell density did not increase with time on the entire scaffold’s surface. This should be overcome by grafting cell-adhesive peptides on the scaffold surface or one of the polymers in the scaffold. This will be done in our future studies.

## STAR★Methods

### Key resources table

**Table:** 

REAGENT or RESOURCE	SOURCE	IDENTIFIER
**Chemicals, peptides, and recombinant proteins**		

Carboxymethyl cellulose	Sigma-Aldrich, Germany	CAS: 9004-32-4
Phosphate buffered saline solution	Sigma-Aldrich, Germany	CAS: 7647-14-5
Streptomycin	Sigma-Aldrich, Germany	CAS: 57-92-1
Penicillin	Sigma-Aldrich, Germany	CAS: 69-57-8
Nanofibrillated cellulose	University of Maine, USA	CAS: 9004-34-6
Citric acid	Carl-Roth, Germany	CAS: 77-92-9
Sodium hydroxide	Sigma-Aldrich, Germany	CAS: 1310-73-2
Advanced Dulbecco’s Modified Eagle’s Medium	ThermoFisher, Germany	Cat. No: 12491015
Fetal Bovine Serum	ThermoFisher, Germany	CAS: 9014-81-7
Carboxymethyl cellulose	Sigma-Aldrich, Germany	CAS: 9004-32-4
Phosphate buffered saline solution	Sigma-Aldrich, Germany	CAS: 7647-14-5
Streptomycin	Sigma-Aldrich, Germany	CAS: 57-92-1
Penicillin	Sigma-Aldrich, Germany	CAS: 69-57-8

**Other**		

Modular Compact Rheometer	Modular Compact Rheometer, Anton Paar	https://www.anton-paar.com/si-en/products/details/rheometer-mcr-102-302-502/
3D printer	BioScaffolder 3.1 (GeSim, Germany)	https://www.bio-strategy.com/site/bio-strategy-nz/files/Supplier_Subs/Gesim_BIOSCAFFOLDER31_web.pdf
Vacuum oven	Vacucell 22; MMM, Munich, Germany	https://www.mmm-medcenter.com/vacucell-62-vacucell-22---evoline47
Field emission scanning electron microscope	Carl Zeiss FE-SEM SUPRA 35 VP electron microscope	https://www.zeiss.com/microscopy/int/products/scanning-electron-microscopes/upgrades/fe-sem.html https://wiki.smfi.unipr.it/dokuwiki/lib/exe/fetch.php?media=lmn:brochure.pdf
Microcomputed tomographer	SkyScan 1275, Bruker μCT	https://www.bruker.com/en/products-and-solutions/microscopes/3d-x-ray-microscopes/skyscan-1275.html
Solid-state NMR spectrometer	Agilent Technologies NMR System 600 MHz NMR spectrometer	http://www.slonmr.si/spectrometers/600_magic.php https://www.agilent.com/cs/library/datasheets/public/5991-3228EN.pdf
Thermogravimetric Analyzer	PerkinElmer (Waltham, MA) TGA 4000	https://resources.perkinelmer.com/lab-solutions/resources/docs/PRD_TGA_4000.pdf
Goniometer	Goniometer system OCA15+ (Dataphysics, Germany)	https://www.dataphysics-instruments.com/Downloads/OCA_V1.3_EN.pdf
Compression testing machine	universal material testing machine (Z020, Zwick Roell, Germany) DMA (Netzsch DMA 242)	https://www.zwickroell.com/products/static-materials-testing-machines/universal-testing-machines-for-static-applications/https://analyzing-testing.netzsch.com/en/products/dynamic-mechanical-analysis-dma/dma-242-e-artemis
Spectrophotometer	Varioskan multiplate reader (ThermoFisher, Germany)	https://spwindustrial.com/thermo-varioskan-flash-multimode-plate-reader-w-software/
Fluorescent microscope	EVOS FL Cell Imaging System, Thermo Fisher Scientific Inc., Germany	https://www.biocompare.com/20180-Microscope-Imaging-Systems/5882825-EVOS-FL-Cell-Imaging-System/

### Resource availability

#### Lead contact

Further information and requests for resources and reagents should be directed to and will be fulfilled by the lead contact, Tamilselvan Mohan (tamilselvan.mohan@tugraz.at).

#### Materials availability

All data supporting the newly fabricated inks and 3D scaffolds can be found within the manuscript and the supplemental information or can be received from the lead contact upon request.

### Method details

#### General materials details

Carboxymethyl cellulose (CMC, sodium salt, degree of substitution (DS)_COOH_ = 0.9, Molecualr weight: 700 kDa), phosphate buffered saline (PBS) (Bioperformace certified, pH 7.4), streptomycin and penicillin were purchased from Sigma-Aldrich, Germany. Nanofibrillated cellulose (NFC, 3 wt.% solid content) was purchased from the University of Maine, Process Development Center, USA. Citric acid (CA ≥ 99.5%) was bought from Carl-Roth, Germany. Advanced Dulbecco’s Modified Eagle’s Medium (ADMEM) and Fetal Bovine Serum (FBS) were purchased from ThermoFisher, Germany. Ultrapure water (Milli-Q system, Millipore, USA; R > 18.18 M Ω cm) was used for the preparation of all samples.

#### Preparation, characterization and evaluation of scaffolds

##### Preparation of composite inks for direct ink writing (DIW)

The following procedure was used to prepare cellulose-based composite inks for DIW (see also Table 1). First, 6 g of CMC was added slowly to citric acid dissolved in water at different concentrations (2.5, 5 and 10 g/g) and stirred with a mechanical laboratory stirrer (IKA EUROSTAR 20) at 150-350 rpm for 40 min. To this, 50 g of NFC (3 wt.%, solid content) was added and stirred with a mechanical stirrer at 200 rpm until no more NFC fibers were seen (ca. 30 min). The inks were covered with an aluminum foil and stored in a refrigerator at 2-8°C until further use. All inks were equilibrated to room temperature prior to the 3D printing experiments.

#### Rheology

The rheological properties of all inks were determined with a Modular Compact Rheometer (MCR 302, Anton Paar, Germany) at 25°C. Viscosity curves were measured from a shear rate of 1 to 100 s^−1^. Frequency sweeps were evaluated at constant strain of 0.1% (in the linear viscoelastic region) in frequency window of 0.1 to 10 s^−1^.

#### Scaffolds fabrication by direct ink writing and freeze-drying

3D printing of the inks (see Table 1) was performed using a BioScaffolder 3.1 (GeSim, Germany). Briefly, a 10 mL polyethylene-based plastic syringe (Nordson, U.K. Limited) with an inner nozzle diameter of 250 μm was used to dispense the inks on a polystyrene petri dish (diameter: 5 cm). Circular scaffolds (radius 7 mm; height: 5-8 mm, number of corners: 100) and cubic shape scaffolds (diameter 25 mm, height 3.5 mm, number of corners: 4) were built in a layer-by-layer fashion. These dimensions were created by using GeSiM Robotics BS3.1/3.2 software. Scaffolds were printed by adjusting the dispensing pressure from 180 kPa – 230 kPa and the distance between strands was kept at 500 μm (minimum distance: 300 μm). The strand height and width were set to 0.2 mm. The printing patterns of each subsequent layer were rotated by 90°, printing speed was 15 mm/s and Z-offset was 0.1 mm.

Scaffolds in the shapes of a cylinder (radius 7 mm, inner radius: 3.5 mm; height 8 mm; number of corners at the edge, 100) and a cuboid were created for mechanical studies (radius 12.5 mm; height 3.5 mm; number of corners: 4). Cylindrical scaffolds (radius, 5 mm; height, 5 mm; number of corners: 100) were prepared for biocompatibility studies, as well as all other studies, which were not previously mentioned (radius, 7 mm; height, 5 mm; number of corners: 100). In this case. the strand height and width, angle of rotation and printing speed were the same as described above.

All printed samples were frozen at −25°C for 48 h in a freezer followed by freeze-drying for 48 h at 10^−3^ mbar and −25°C

#### Crosslinking and neutralization

After freeze-drying, the dry scaffolds were chemically crosslinked by dehydrothermal (DHT) treatment (Drexler and Powell, 2011; Haugh et al., 2009). Scaffolds were placed in a glass container, covered completely with aluminum foil and kept in a vacuum oven (Vacucell 22; MMM, Munich, Germany) at 100 mbar at 120°C for 24 h. The crosslinked scaffolds were immersed in 10 mL of 0.1 M NaOH solution and stirred for 20-60 min. Subsequently, the scaffolds were immersed in 200 mL water (pH 7.4) for 24 h with constant stirring. Following this, each scaffold was rinsed three times with water to remove excess or non-crosslinked citric acid. Afterwards, the scaffolds were placed onto a filter paper (115A-type) and air-dried at ambient condition. The crosslinked, neutralized and air-dried scaffolds are named as “Ink***x***/***y/N***” where ***x*** is the concentration of citric acid in wt.%, y is the temperature in °C and N indicates the applied neutralization.

Furthermore, all neutralized wet scaffolds were immersed in 10 mL of biofluid (ADMEM +5% FBS +100 IU mL^−1^ penicillin and 0.1 mg mL^−1^ streptomycin) containing phenolic red for 30 min at 37°C with constant stirring. The color change of the biofluid was observed.

#### Morphology and porosity analysis

##### Field emission scanning electron microscopy (FESEM)

The morphology of lyophilized scaffolds was analyzed by FESEM (Carl Zeiss FE-SEM SUPRA 35 VP electron microscope). Prior to imaging, all samples were pressed onto a double-sided carbon adhesive tape (SPI 116 Supplies, USA). No sputtering was performed on the sample surfaces. The freeze-dried scaffolds were immersed into liquid nitrogen and fractured to analyze the cross-section of the samples. The images were recorded with an acceleration voltage of 1 kV at room temperature. For the image analysis of the SEM images the software ImageJ/FIJI 1.53c (National Institute of Health, USA) (Schindelin et al., 2012) was used by generating a binary image and subsequently using the inbuild particle measurements. Pores smaller than 10 pixel2 (approx. 63 μm^2^) were excluded from the calculation to avoid miscounts from single pixels and artifacts.

##### Microcomputed tomography (μCT)

Samples were analyzed in a X-ray microcomputed tomography (μCT) equipment from SkyScan 1275 (Bruker μCT, Kontich, Belgium) with penetrative X-rays of 31 kV and 216 μA, in high resolution mode with a pixel size of 8 μm, 92 ms of exposure time, 25 of frame averaging, 0.20 deg of rotation step and 360° of rotation. NRecon (v.1.7.3.1 software, Bruker, Kontich, Belgium) and CTVox (v.3.3.0 r1403 software, Bruker, Kontich, Belgium) softwares were used for 3D-reconstruction and CTAn software (v.1.17.7.2 software, Bruker, Kontich, Belgium) was used in morphometric analysis (e.g., porosity values and pore size distribution), in which the images were segmented and analyzed. Herein, the thresholding or image binarization/segmentation was performed using a global thresholding technique for each sample which employs a fixed range of greyscales (lower and upper scales set at 69 and 255) for both foreground (white) and the pixels outside of the range area, which are set as the background (black).

##### Wet (μCT) measurements

Samples were immersed in MilliQ-water for 24, 48 and 72 h, respectively. Afterwards, the sample were analyzed with penetrative X-rays of 35 kV and 231 μA, in high resolution mode with a pixel size of 13 μm, 30 ms of exposure time, 25 of frame averaging, 0.20 deg of rotation step and 360° of rotation.

#### Solid-state nuclear magnetic resonance (NMR)

Solid-state NMR spectra were acquired on Agilent Technologies NMR System 600 MHz NMR spectrometer equipped with 1.6 mm Triple Resonance HXY FastMAS probe. Larmor frequency of proton and carbon nuclei was 599.41 and 150.72 MHz, respectively. 1H and 13C NMR chemical shifts are reported relative to TMS (*δ* 0.0 ppm). Samples were spun at 20,000 Hz.

#### Thermogravimetric analysis (TGA)

TGA was performed on a PerkinElmer (Waltham, MA) TGA 4000 thermal analysis instrument in a nitrogen atmosphere (20 mL min^−1^) from 40 to 900°C with a heating rate of 10 °C min^−1^ using an Al_2_O_3_ crucible without a lid. For data evaluation, the Pyris software, version 10.02.0468, was used.

#### Contact angle measurements

Static water contact angle (SWCA) measurements were carried out using ultra-pure water on a goniometer system OCA15+ (Dataphysics, Germany). All measurements were carried out at ambient temperature with a drop volume of 3 μl.

#### Analysis of swelling capacity and weight loss

The swelling kinetics of the Ink***x***/*y****/****N* scaffolds in biofluid (ADMEM +5% FBS +100 IU mL^−1^ penicillin and 0.1 mg mL^−1^ streptomycin) were investigated using a gravimetric method (Kurečič et al., 2019; Mohan et al., 2020). The dried cylinder-shaped scaffolds (*r* = 7 mm, *h* = 5 mm) were weighed (initial weight, W_0_), immersed in 5 mL biofluid (pH 7.4) at 37°C. At predetermined time intervals (W_t_), the scaffolds were removed from the biofluid, wiped dry carefully by a filter paper only on the surface and weighed again. The swelling capacity at time *t* was calculated using Equation (1).

(Equation 1) $$\text{Swelling capacity }\left(\text{\%}\right)=\frac{\text{Wt}-\text{Wo}}{\text{Wo}}\times 100$$

To determine the weight loss upon contact with bioliquid, the scaffolds (initial weight, W_0_) were placed in a beaker with 5 mL of biofluid at 37°C and stirred at 200 rpm. At predetermined intervals, the scaffolds were removed from the biofluid, washed three times with water and lyophilized as mentioned above. The remaining weight (RW) of the scaffolds was calculated as follows:

(Equation 2) $$\text{RW }\left(\text{\%}\right)=\frac{\text{Wt}}{\text{Wo}}\times 100,$$

where W_t_ is the dry weight of the scaffold at a predetermined time. All experiments were repeated at least three times and arithmetic mean values and standard deviation of triplicates were calculated.

#### Mechanical strength analysis

##### Compression measurements

Static compression testing was performed for the prepared scaffolds. Before the measurements, the scaffolds were completely immersed in biofluid for approx. 2 h at ambient temperature. After equilibration, the diameter of the cylindric scaffolds (approx. dimensions 14 mm × 9 mm) was measured with a digital caliper. The samples were tested with universal material testing machine (Z020, Zwick Roell, Germany) equipped with a 500 N load cell was used for the unconfined compression test. The strain rate was set to 2.4 mm min^−1^ and samples were compressed to 40% of the initial height. After compression, the elastic relaxation to 5% of the maximum stress (at 40%) at a relaxation rate of 2.4 mm min^−1^ was determined. The elastic modulus was calculated from the slope in the initial stage of the stress-strain curve (2-8% strain) and the compressive strength was extracted at 30% strain for all samples (at least three specimens per sample) according to the literature (Dobaj Štiglic et al., 2021; Mohan et al., 2020).

Dynamic compression tests were performed with a dynamic mechanical analyzer (DMA) (Netzsch DMA 242) in compression mode. Prior to analysis, the samples were equilibrated in CCM according to the sample preparation for static compression. The samples were cut in small cubes (approx. 5 × 5 × 2.5 mm) and studied at a frequency range from 0.2 to 100 Hz at 20°C. Testing limits on amplitude, maximum dynamic force, and static constant force were set as 32.4 μm, 7 N, and 1 N, respectively.

#### *In vitro* biocompatibility

##### MTT assay on sample extracts

The influence of the scaffolds on cell viability was evaluated via the reduction reaction of the tetrazolium salt MTT (3(4,5 dimethylthiazolyl-2)-2,5-diphenyltetrazolium bromide), purchased from Sigma Aldrich, Germany. Scaffolds were exposed to the UV light for 30 min for sterilization. The samples were soaked into 3 mL of Advanced DMEM supplemented with 5% FBS and incubated for 24 h at 37°C in an atmosphere containing 5% CO_2_. The human bone tissue derived osteoblast cells (hFOBs, ATCC, UK) (10,000 cells/well) were seeded into a 96-well microtitre plate with a final volume of 100 μL of Advanced DMEM with 5% FBS. The material samples (supernatants of the starting samples) were added to the cells after 24 h of incubation at 37°C in 5 wt.% CO_2_ in four parallels and in several dilutions (undiluted, 1:2, 1:4, 1:8 and 1:16). As control, Advanced ADMEM and 5% FBS was added to the cells. After 24 h of treatment, cell viability was determined using the MTT test (Maver et al., 2018; Stergar et al., 2017). For this purpose, 10 wt.% reagent was added to the medium and discarded after 4 h. Then, 100 μL of DMSO was added and after 5 min, the absorbance was measured spectrophotometrically at 570 nm using the Varioskan multiplate reader (ThermoFisher, Germany).

##### XTT and live/dead assays on 3D printed scaffolds

To get further insight into the cell viability after their exposure to the scaffolds, the so-called “cell attachment or direct exposure test” was performed with the scaffold Ink**2.5**/120°C/N. Two types of methods were used, the XTT assay and the Live/dead assay. Prior to performing the tests, the samples were sterilized under UV light for 30 min. Samples were then placed in duplicate in P24 microtiter plates and filled them with 1.5 mL of Advanced DMEM medium containing 5% FBS. After 24 h of incubation at 37°C and 5% CO_2_, the medium was pipetted off, and the cells were seeded at a density of 20,000 cells per scaffold (volume 30 microliters per scaffold). After incubation at 37°C and 5% CO_2_ for four hours, the scaffolds were poured with 1 ml of Advanced DMEM medium containing 5% FBS. Then, the scaffolds were incubated again under controlled atmosphere in an incubator at 37°C and 5% CO_2_. The medium was changed every 3 days and the tests were performed at three time points. From here on, the testing method was different for the two used methods.

The Live/Dead assay (Sigma-Aldrich, Germany; it includes Calcein-AM, which dyes only living cells (green colour), and propidium iodide (PI), which dyes only dead cells (the dye cannot penetrate intact cell membranes) was used, and served at the same time as an additional proof (apart from the above described quantitative MTT and the following XTT assay) of the prepared materials’ biocompatibility towards the target cells tested (human bone-derived osteoblasts, hFOB 1.19 (ATCC CRL-11372), ATCC, UK). After incubation, the tested samples were rinsed with PBS (Sigma-Aldrich, Germany), followed by incubation in 4 mM calcein-AM and 2 mM PI solutions, respectively. Both solutions were prepared in PBS. The sample incubation with the Live/Dead kit was performed for 40 minutes at 25°C. The final step in the process, was another washing of the samples with PBS. After the mentioned procedure, the samples were slide upside down into a new wells filled with 500 μL of PBS. The samples were observed under a fluorescent microscope at 10× magnification (EVOS FL Cell Imaging System, Thermo Fisher Scientific Inc., Germany).

After 6 days of incubation, the XTT assay was performed according to the manufacturer’s instructions. The XTT reagent (500 μl) was added to the medium (1 ml), and the scaffolds were incubated for 4 hours at 37°C, 5 wt.% CO_2_. Then, 100 μl of the medium was pipetted onto a P96 microtiter plate, and the absorbance was measured at 450 and 650 nm using the Varioskan spectrophotometer (Thermo Fisher Scientific, Germany). When interpreting the results, these were considered together with the Live/Dead assay results, where cells were clearly visible in the case of the Ink**2.5**/120°C/N.

### Quantification and statistical analysis

All numerical values are given as arithmetic mean and standard deviation. Statistical analysis was performed using either SPSS Statistics 25 (IBM Corp. Armonk, NY, USA). By using the Shapiro-Wilk test it was determined that all experimental data was normally distributed, allowing the use of one-way ANOVA. p-values <0.05 were considered to be statistically

## Data Availability

• Any additional information required to reanalyze the data reported in this paper is available from the lead contact upon request. • Data: All data reported in this paper will be shared by the lead contact upon request. • Code: This paper does not report original code.
