# Supplementary material for: Organic acid cross-linked 3D printed cellulose nanocomposite bioscaffolds with controlled porosity, mechanical strength, and biocompatibility
Source: iScience. 2022 Apr 16;25(5):104263. doi: 10.1016/j.isci.2022.104263 (PMC9062678; doi:10.1016/j.isci.2022.104263)
Supplement: Document S1. Figures S1–S10 [file mmc1.pdf]

iScience, Volume *25*

Supplemental information

Organic acid cross-linked 3D printed cellulose nanocomposite bioscaffolds with controlled porosity, mechanical strength, and biocompatibility

Andreja Dobaj Štiglic, Fazilet Gürer, Florian Lackner, Doris Bračič, Armin Winter, Lidija Gradišnik, Damjan Makuc, Rupert Kargl, Isabel Duarte, Janez Plavec, Uros Maver, Marco Beaumont, Karin Stana Kleinschek, and Tamilselvan Mohan

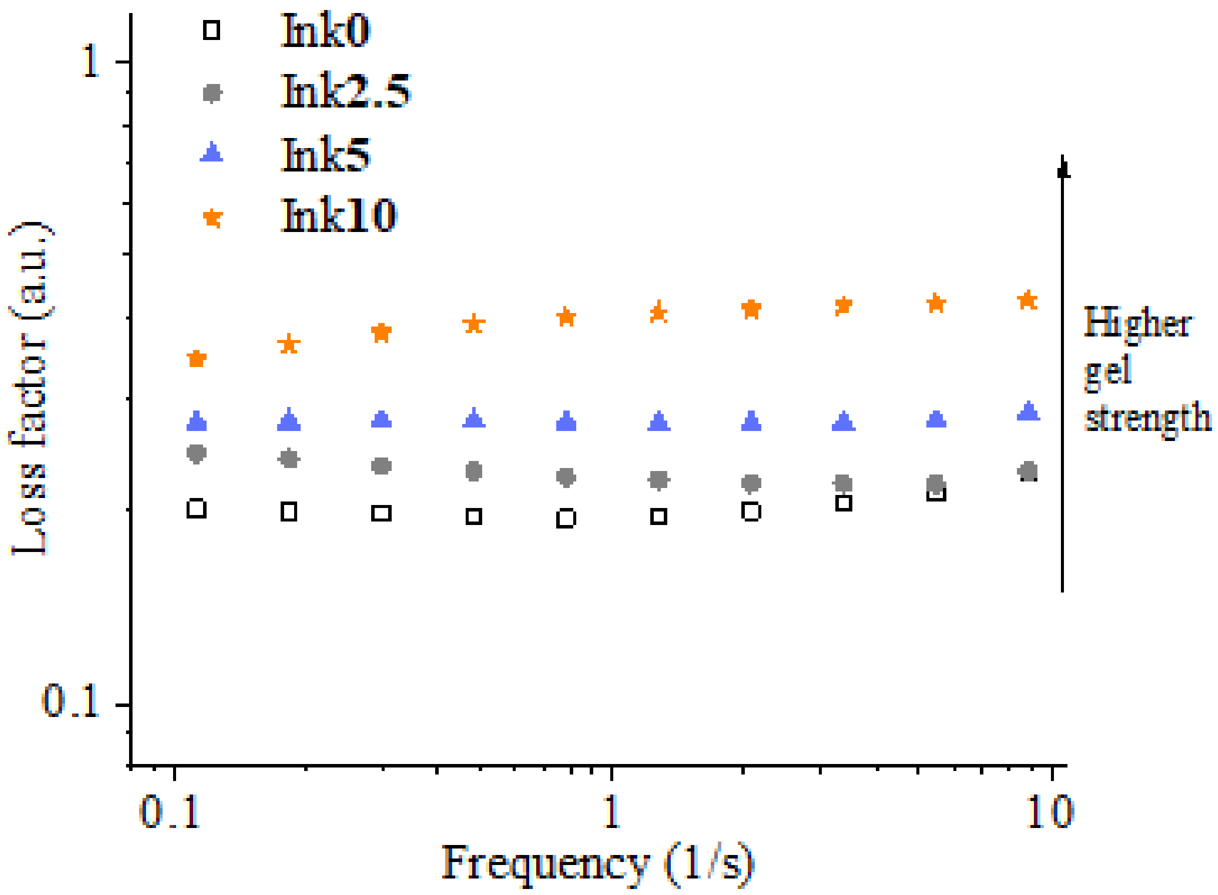

Figure S1. Dependency of the loss factor from the frequency determined by rheology measurements, (related to **Figure 1**).

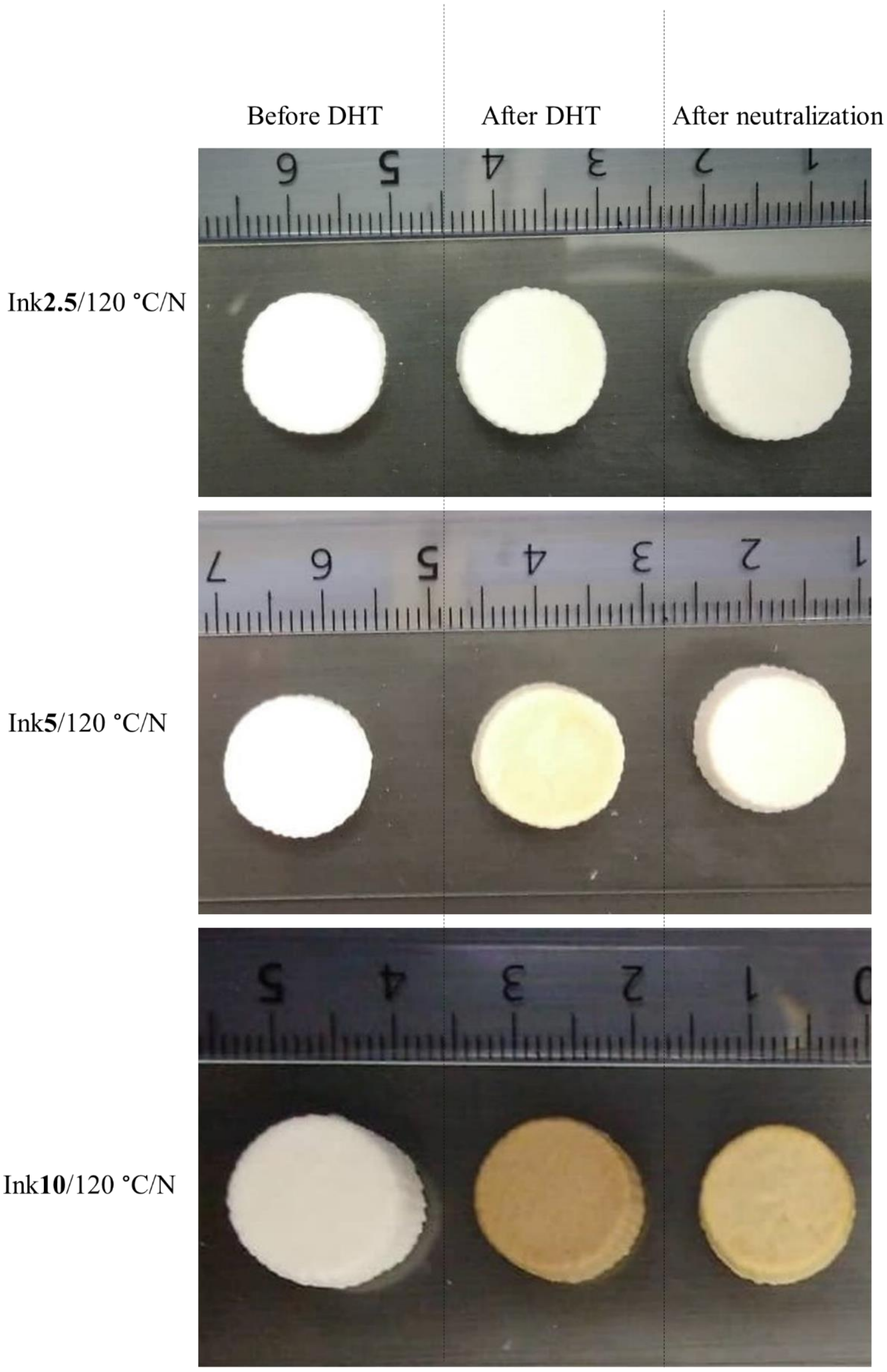

Figure S2. Photos of NFC/CMC scaffolds crosslinked with different concentrations: before and after dehydrothermal (DHT) treatment and after neutralization. Top: Ink**2.5**/120 °C/N, Middle: Ink**5**/120 °C/N, Bottom: Ink**10**/120 °C/N, (related to **Figure 2**)

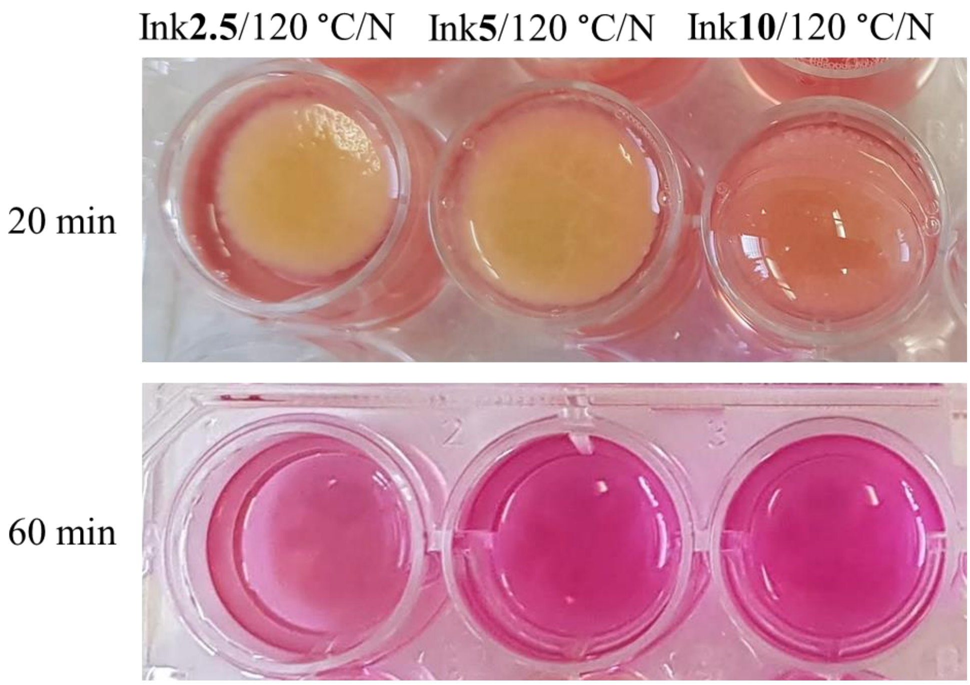

Figure S3. Photo images of citric acid crosslinked scaffolds of NFC/CMC after immersion in biofluid at 20 and 60 min, (related to **Figure 2)**.

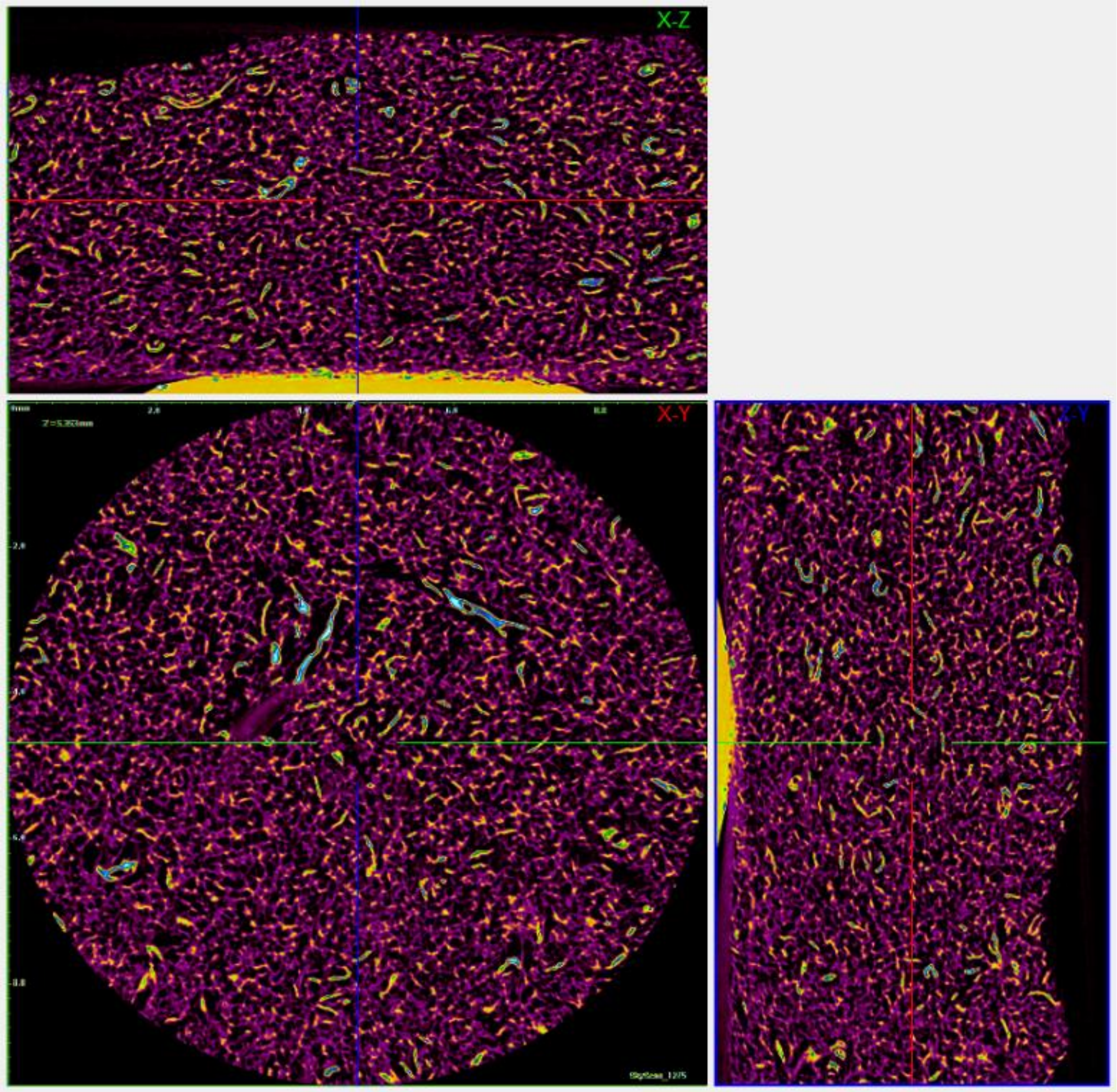

Figure S4. The 2D top and side views of Ink**0**/120 °C (non-crosslinked), (related to **Figure 4**).

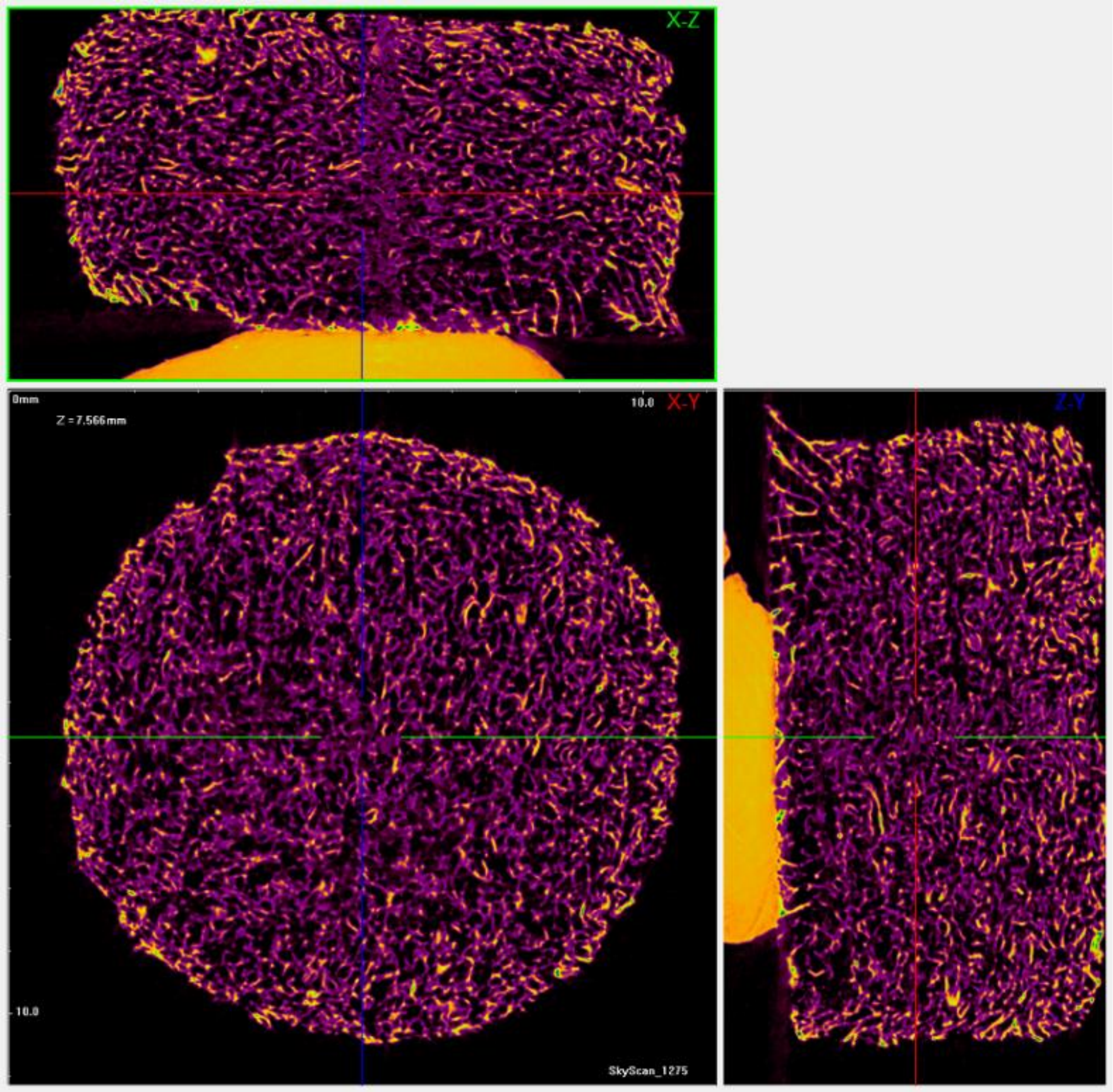

Figure S5. The 2D top and side views of Ink**2.5**/120 °C/N (citric acid-crosslinked), (related to **Figure 4**).

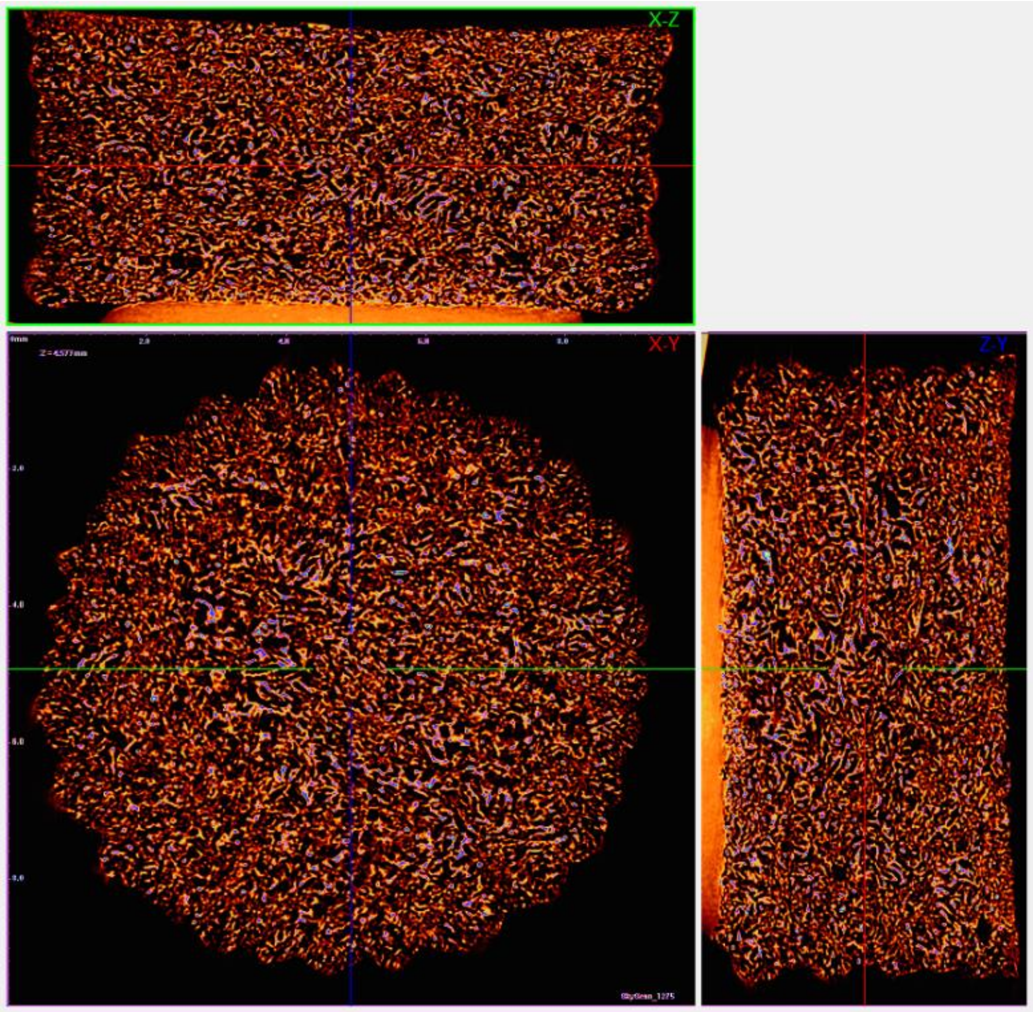

Figure S6. The 2D top and side views of Ink**10**/120 °C/N (citric acid-crosslinked), (related to **Figure 4**).

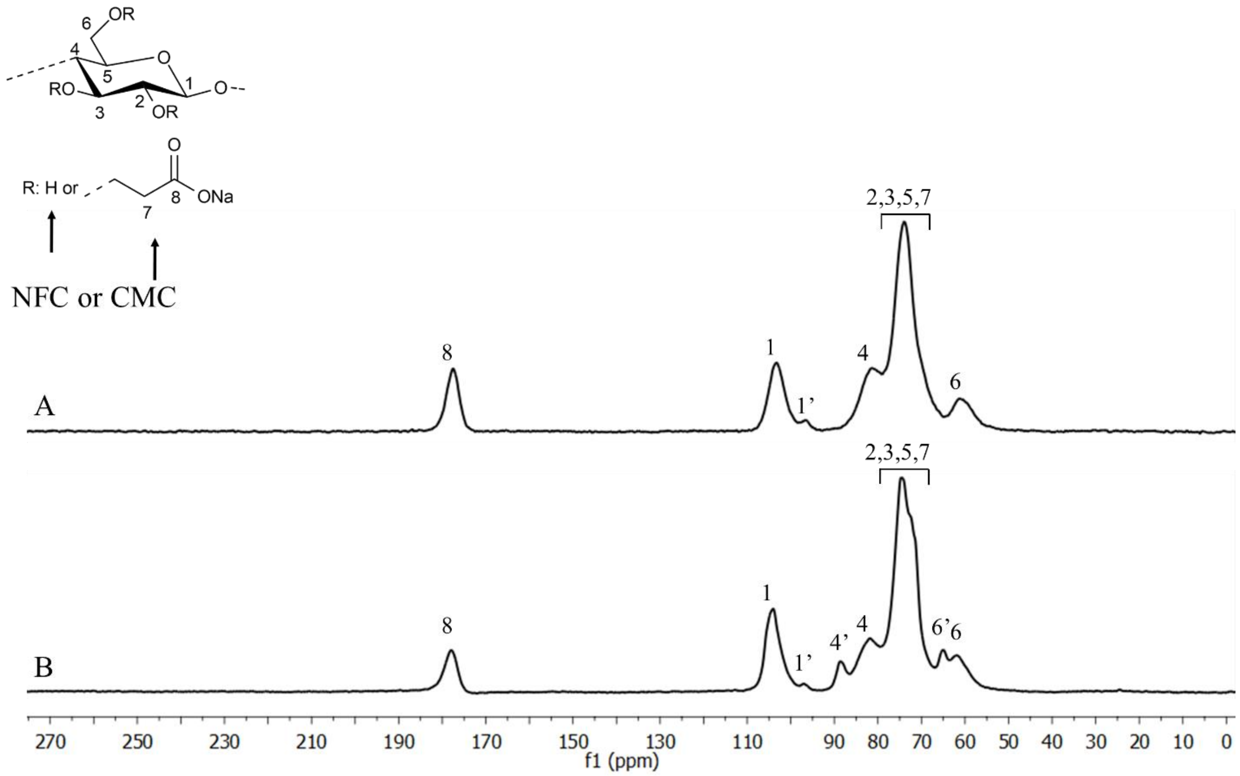

Figure S7. ^{13}C-solid state NMR spectra. (A). Neat carboxymethyl cellulose. (B) Ink**0**/120 °C (non-crosslinked), (related to **Figure 6**).

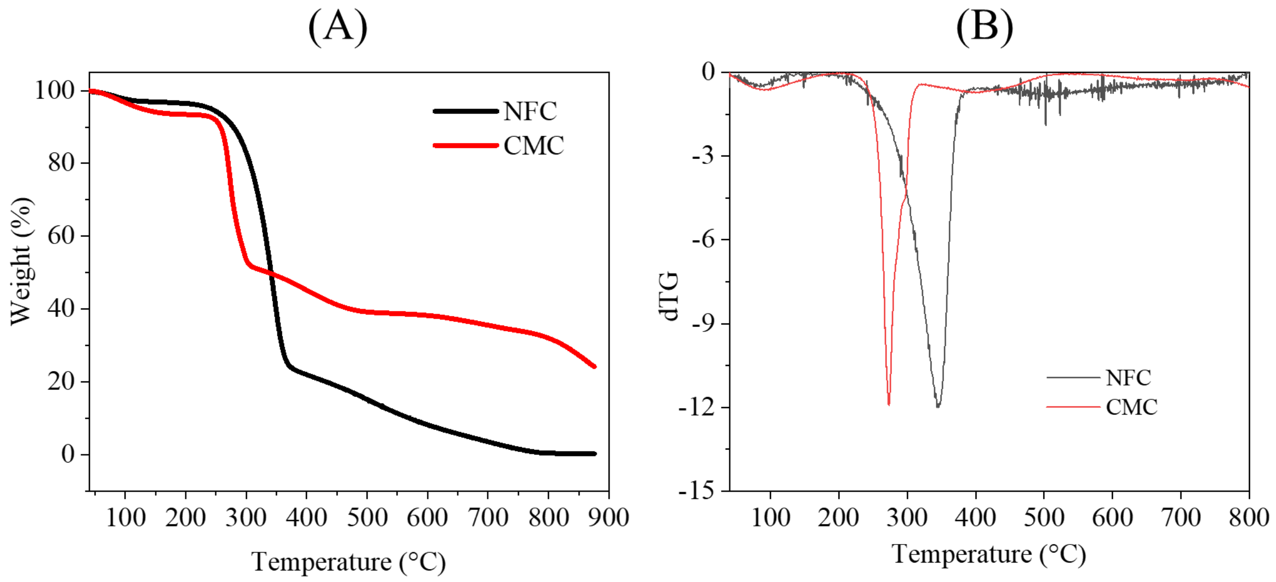

Figure S8. **Thermal stabilities of crosslinked and non-crosslinked scaffolds.** (A) TGA curves of neat NFC and CMC. (B) dTG curves fo neat NFC and CMC, (related to **Figure 8**).

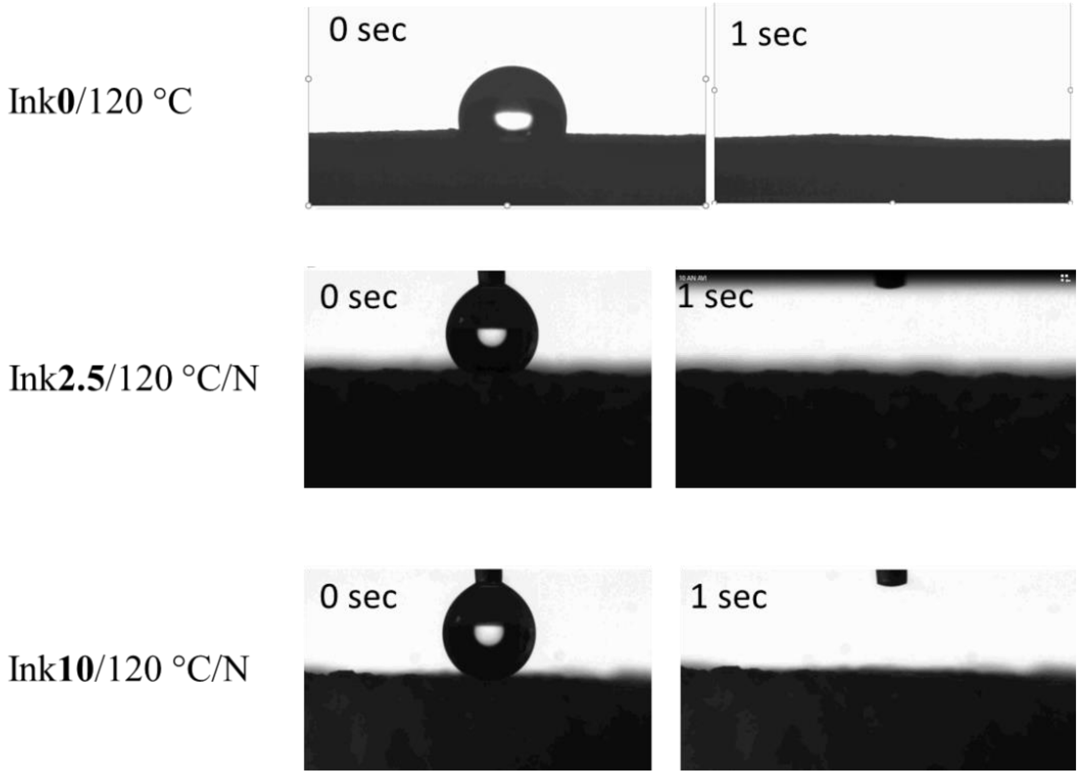

Figure S9. Photo images of water contact angle measurements of non-crosslinked and citric acid crosslinked scaffolds, (related to **STAR+METHODS**, contact angle measurements).

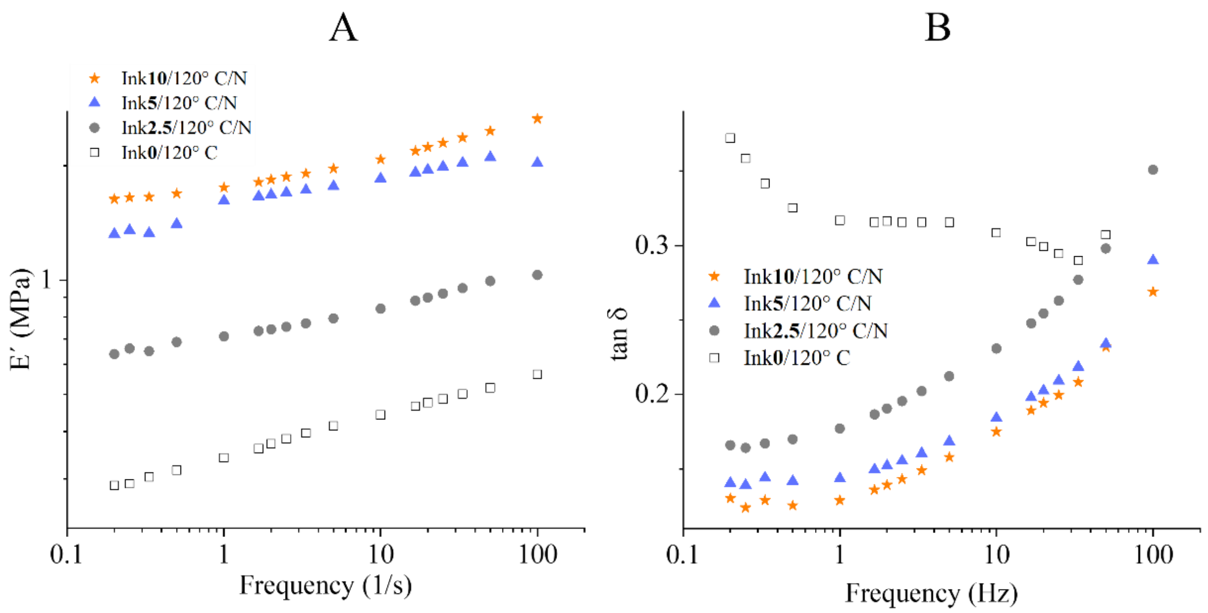

Figure S10. Dynamic mechanical analysis of the hydrogels. (A) storage modulus (E'). (B) damping factor (tanδ) of wet samples. The different damping behavior of the non-crosslinked scaffold (Ink**0**/120 °C) can be explained through occurring plastic deformation and densification, (related to **Figure 9**).
